# A novel α-conopeptide Eu1.6 inhibits N-type (Ca_V_2.2) calcium channels and exhibits potent analgesic activity

**DOI:** 10.1038/s41598-017-18479-4

**Published:** 2018-01-17

**Authors:** Zhuguo Liu, Peter Bartels, Mahsa Sadeghi, Tianpeng Du, Qing Dai, Cui Zhu, Shuo Yu, Shuo Wang, Mingxin Dong, Ting Sun, Jiabin Guo, Shuangqing Peng, Ling Jiang, David J. Adams, Qiuyun Dai

**Affiliations:** 1 Beijing Institute of Biotechnology, Beijing, 100071 China; 2 Health Innovations Research Institute, RMIT University, Melbourne, Victoria, 3083 Australia; 3 Illawarra Health and Medical Research Institute (IHMRI), University of Wollongong, Wollongong, NSW 2522 Australia; 4 Key Laboratory of Magnetic Resonance in Biological Systems, National Center for Magnetic Resonance in Wuhan, State Key Laboratory of Magnetic Resonance and Atomic and Molecular Physics, Wuhan Institute of Physics and Mathematics, Chinese Academy of Science, Wuhan, 430071 China; 5 Institute for Disease control and Prevention, Beijing, 100071 China

## Abstract

We here describe a novel α-conopeptide, Eu1.6 from *Conus eburneus*, which exhibits strong anti-nociceptive activity by an unexpected mechanism of action. Unlike other α-conopeptides that largely target nicotinic acetylcholine receptors (nAChRs), Eu1.6 displayed only weak inhibitory activity at the α3β4 and α7 nAChR subtypes and TTX-resistant sodium channels, and no activity at TTX-sensitive sodium channels in rat dorsal root ganglion (DRG) neurons, or opiate receptors, VR1, KCNQ1, L- and T-type calcium channels expressed in HEK293 cells. However, Eu1.6 inhibited high voltage-activated N-type calcium channel currents in isolated mouse DRG neurons which was independent of GABA_B_ receptor activation. In HEK293 cells expressing Ca_V_2.2 channels alone, Eu1.6 reversibly inhibited depolarization-activated Ba^2+^ currents in a voltage- and state-dependent manner. Inhibition of Ca_V_2.2 by Eu1.6 was concentration-dependent (IC_50_ ~1 nM). Significantly, systemic administration of Eu1.6 at doses of 2.5–5.0 μg/kg exhibited potent analgesic activities in rat partial sciatic nerve injury and chronic constriction injury pain models. Furthermore, Eu1.6 had no significant side-effect on spontaneous locomotor activity, cardiac and respiratory function, and drug dependence in mice. These findings suggest α-conopeptide Eu1.6 is a potent analgesic for the treatment of neuropathic and chronic pain and opens a novel option for future analgesic drug design.

## Introduction

The mere abundance of marine animals evolving large repertoires of neuroactive peptides underlies the substantial interest in the therapeutic potential that rests on natural products particularly from the venom of marine predatory fish-hunting cone snails (*Conus* species)^1–3^. More than 800 known *Conus* species have built a large genetic library with >200,000 neuroactive ligands of which less than 0.1% have been pharmacologically characterized^4–6^.

Many of these uncharacterized neuroactive peptides are currently under investigation for novel treatments in Parkinson’s disease, multiple sclerosis, lung cancer and intractable chronic pain conditions^1,3,7^. Omega (ω)-conotopeptide MVIIA (Prialt) from *Conus magus* was the first *Conus* peptide to be approved by U.S. and European regulatory agencies for the treatment of chronic intractable pain in patients who do not respond to conventional opioid therapies^8^. However, the overall success of Prialt failed to be realized because of its neurological and cardiovascular side effects and its inconvenient method of application (intrathecal administration, I.T.)^9–11^. The smaller α-conopeptides (12–16 amino acids) of the A-superfamily exhibit a distinct pharmacological antagonism with respect to their high potency at inhibiting muscle or neuronal nAChRs^12–16^. Several α-conopeptides, including Vc1.1, Rg1A, PeIA, and AuIB, have been reported to alleviate neuropathic pain by inhibiting N-type calcium channels via G protein-coupled GABA_B_ receptor activation^17–21^.

The physiological role of N-type (Ca_V_2.2) calcium channels in neuronal excitation and synaptic transmission has been well described and accentuates them as attractive targets for clinical pain intervention^5–7,9,22–27^. Voltage-gated calcium channels are comprised of a pore-forming α_1B_ subunit which activates (open) upon membrane depolarization to allow a brief and local Ca^2+^ influx into the cell, and then inactivates before returning to the resting state with membrane repolarization. This intrinsic and highly conserved mechanism is supported by one of the four known *β* subunits (*β*1 − *β*4) and a α_2_δ subunit (*α*_2_*δ*1 − *α*_2_*δ*4) which aid in a chaperone-like manner to modulate channel expression as well as biophysical and pharmacological channel properties^28,29^. The *α*_1B_ and *α*_2_*δ* subunits have become of increasing interest as pharmacological targets to alleviate chronic pain conditions^8,9,30,31^.

In the present study, we describe a novel Ca_V_2.2 inhibitor from the ivory cone snail (*C*. *eburneus*). Unlike other α-conopeptides that target neuronal nAChRs or GABA_B_ receptors, Eu1.6 potently inhibits Ca_V_2.2 with only a weak interaction at α3β4 and α7 nAChRs subtypes. Eu1.6 inhibited >30% of the high voltage-activated (HVA) calcium current in mouse DRG neurons and a detailed electrophysiological characterization of the inhibition of human Ca_V_2.2 channels was carried out using a recombinant HEK293 cell expression system. Eu1.6 reversibly inhibited Ca_V_2.2-mediated Ba^2+^ currents in a concentration-dependent manner and steady-state inactivation was enhanced at hyperpolarized membrane potentials. Most importantly, Eu1.6 exhibited analgesic activity in rat partial sciatic nerve injury (PNL) and chronic constriction injury (CCI) models at doses of 2.5–5.0 μg/kg, and was more potent than a combination of morphine (5 mg/kg) and gabapentin (100 mg/kg). To our knowledge, this is the first identified α-conopeptide to directly inhibit Ca_V_2.2 channels and display potent analgesic activity.

## Results

### Cloning, synthesis and folding of Eu1.6

**Figure 1:**
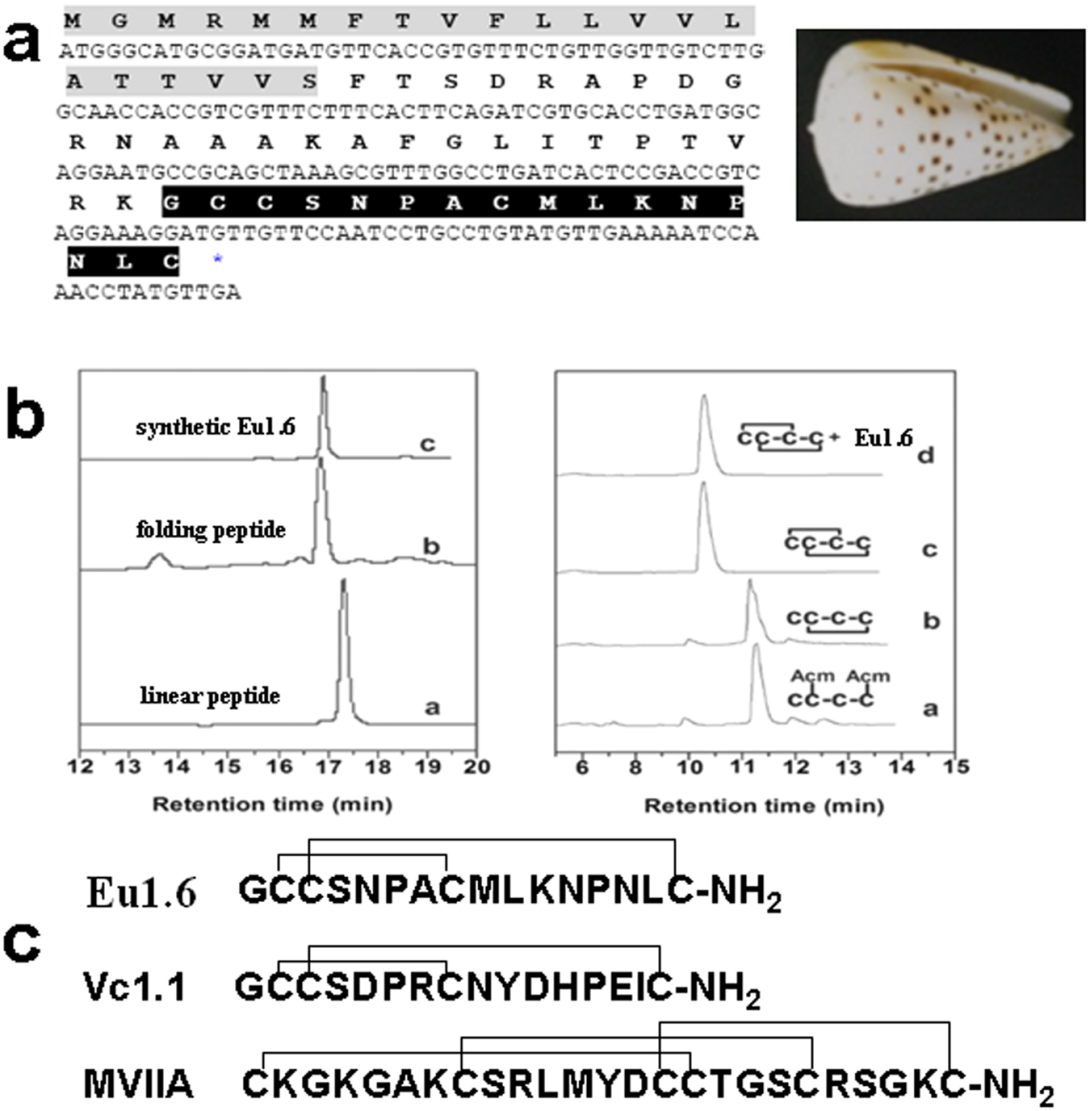
Cloning, synthesis and folding of α-conopeptide Eu1.6. (**a**) The cDNA sequence and predicted translation product of Eu1.6 (above). The signal peptide and mature toxin are shaded. The signal peptide is shown in gray, the propeptide is underlined, and the mature peptide is shown with white text on a blackground. HPLC analyses of the folded products of linear Eu1.6 and its Acm derivatives. Photograph of *C*. *eburneus* shell (right). (**b**) One-step oxidative folding of Eu1.6 (left). Traces from bottom to top: (a) linear peptide; (b) one-step oxidized products; (c) purified product. Determination of the disulfide bond connectivity of Eu1.6 (right). Traces from bottom to top: (a) Linear peptide with Acm modification at Cys 2 and Cys 4; (b) the first oxidized product; (c) The second oxidized product and (d) Co-elution of the two-step folding products and one-step folding products. Samples were applied to a Calesil ODS-100 C_18_ column (4.6 mm × 250 mm) and eluted with a linear gradient of 0~1 min, 5–10% B (B is acetonitrile (0.1% TFA); 1~25 min, 10~50% B; 25~28 min, 50~95% B at a flow rate of 1 ml/min, 214 nm of wavelength. (**c**) Comparison of the primary structure of Eu1.6 with other α-conotoxins that act on nAChRs and GABA_B_ receptor/Ca_V_2.2.

Classification of the conopeptide Eu1.6 was based on ER signal sequences of the precursors (gene superfamily) and Cys pattern of the mature peptide (Cys frameworks)^32^. As an initial strategy, peptide sequences were cloned from the venom glands of living cone snails of *Conus eburneus* harvested near Hainan in the South China Sea (Fig. 1a). A novel conopeptide precursor was cloned from *C*. *eburneus* by 3′ RACE using the conserved signal peptide sequence found in the A-superfamily conopeptides. The mature toxin sequence was predicted as GCCSNPACMLKNPNLC-NH_2_ with a cysteine pattern of CCX_4_CX_7_C (Fig. 1c), suggesting this peptide belongs to the α-4/7 family of conopeptides. In accord with conventional conopeptide nomenclature^32^, the cloned sequence that encodes the novel α-conopeptide was designated as Eu1.6. This cDNA sequence has been submitted to GenBank (accession number HQ446467).

The Eu1.6 linear peptide folded well and formed a major peak (Fig. 1b). The folded peptide was then purified by semi-preparative HPLC. The final synthesized Eu1.6 was further assessed using analytical reversed-phase HPLC, and its purity was greater than 98% with the expected molecular weight. HPLC results of one-step and two-step folding of the acetamidomethyl (Acm)-protected linear peptides are shown in Fig. 1b. The retention time of the synthesized Eu1.6 in the one-step folding was identical to that of Eu1.6, with a disulfide connectivity of I-III, II-IV forming in the two-step oxidation process Fig. 1b,d, indicating the disulfide bond connectivity of Eu1.6 was I-III, II-IV.

### NMR solution structure of Eu1.6

**Figure 2:**
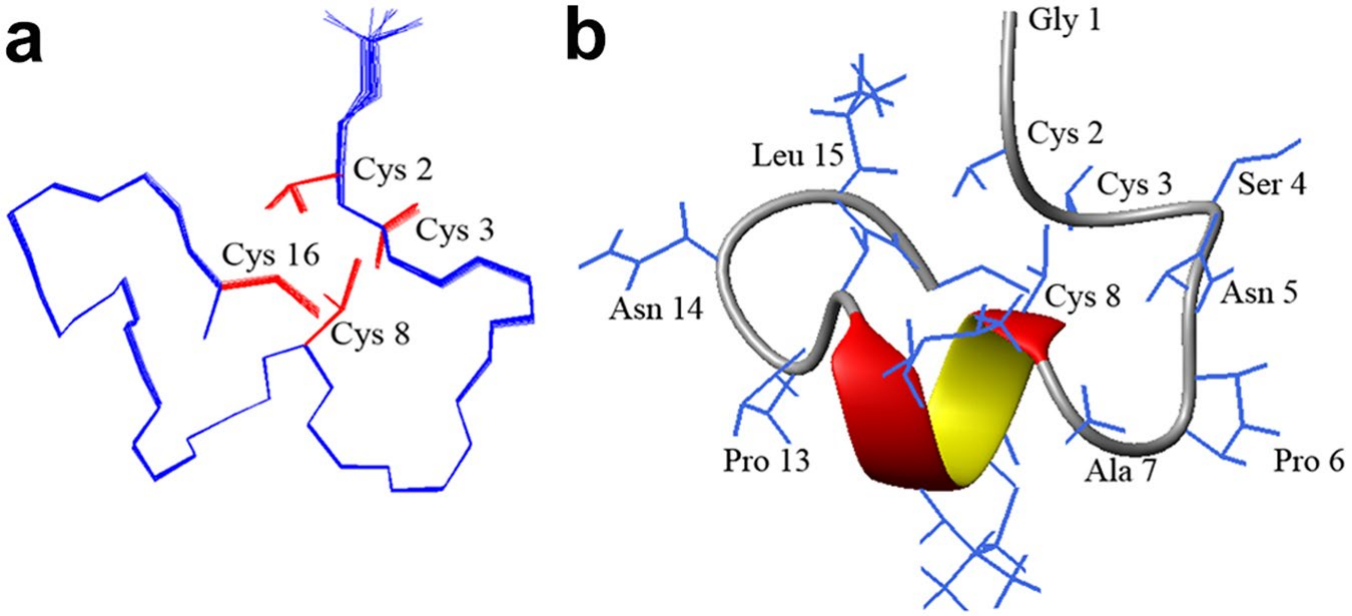
NMR solution structure of Eu1.6. (**a**) Backbone ensemble of 20 lowest energy structures. The side chains of Cys residues are shown in red. (**b**) Ribbon representation of the closest-to-mean structure.

The structural statistics for Eu1.6 are given in Supp. Table S1. The ensemble of the 20-lowest energy structures is shown in Fig. 2a and the ribbon representation is shown in Fig. 2b. The Eu1.6 peptide contains a short α–helix starting from Cys^8^ to Lys^11^. The rest of the peptide is stabilized by two disulfide bonds, Cys^2^-Cys^8^ and Cys^3^-Cys^16^. The loops are well defined and hydrogen bonds can be observed between Ser^4^ NH and Cys^3^ O, Cys^8^ NH and Asn^5^ O, Asn^12^ NH and Cys^8^ O, Cys^16^ NH and Asn^12^ O.

### Eu1.6 exhibits potent analgesic activity in rat PNL and CCI models

**Figure 3:**
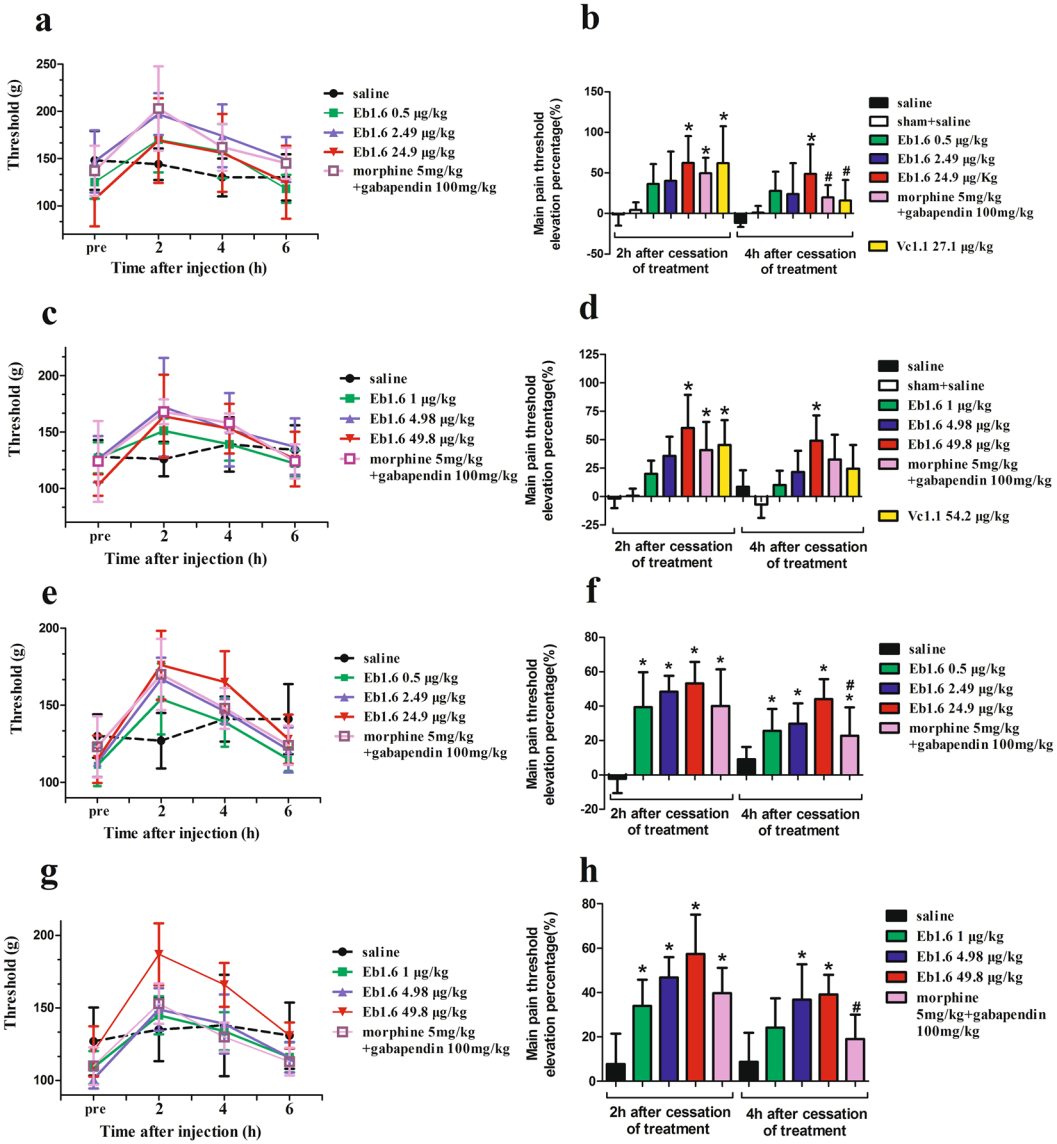
Analgesic activity of Eu1.6 in rat PNL and CCI pain models. (**a**,**b**,**e**,**f**) PNL and CCI rats (n = 8/group) were treated with sterile saline (i.m.), Eu1.6 (0.5, 2.49 and 24.9 μg/kg, i.m.), Vc1.1 (27.2 μg/kg, i.m.), or a combination of morphine (5 mg/kg), subcutaneous injection (sc) and gabapentin (100 mg/kg, intragastric administration (i.g.). Sham rats (n = 8) were treated with sterile saline (i.m.). (**c**,**d**,**g**,**h**) PNL and CCI rats (n = 8/group) were treated with sterile saline (i.v.), Eu1.6 (1, 4.98 and 49.8 μg/kg, i.v.) or the combination of morphine (5 mg/kg, sc) and gabapentin (100 mg/kg, i.g.). The LHS graphs shows the mean pain threshold at pre-injection and 2, 4 and 6 h following treatments (**a,c,e,g**), whereas the RHS bar  graphs show the corresponding elevation percentage at 2 h and 4 h following treatments (**b,d,f,h**). The different groups were analyzed by one-way ANOVA followed by the LSD test for multiple comparison tests at a 0.05 level of significance. *p < 0.05 vs. saline, ^#^p < 0.05 Eu1.6 (low dose group) *vs*. Vc1.1 or the combined group of morphine and gabapentin.

Given that some α-conopeptides have been shown to exhibit analgesic activity, Eu1.6 was evaluated initially using a rat PNL model^18,19^. Eu1.6 displayed potent analgesic activity in this analgesic model whereby at 2 hr after ipsilateral muscular injection (i.m.) of different doses of Eu1.6 (0.5 μg/kg (0.3 nmol/kg), 2.49 μg/kg (1.5 nmol/kg) and 24.9 μg/kg (15 nmol/kg), the pain threshold was 170 ± 34.6 g, 197 ± 22.1 g, 169 ± 44.8 g (n = 8), respectively (Fig. 3a), higher than the starting value (125 ± 17.6 g, 147 ± 33.0 g, 107 ± 28.7 g; n = 8). The elevation percentage of rat pain threshold ((pain threshold − pain threshold of pre-experiment)/pain threshold of pre-experiment × 100%) at 2 hr after 24.9 μg/kg (15 nmol/kg) of Eu1.6 administration was 62% (Fig. 3b), similar to that observed for α-conotoxin Vc1.1 (27.1 μg/kg (15 nmol/kg), and morphine (5 mg/kg) with gabapentin (100 mg/kg) positive control group (62% and 50%, respectively). However, 4 hr after administration, the pain threshold elevation percentage decreased (49%, 24.9 μg/kg), but was still higher than Vc1.1 and the morphine plus gabapentin group (16% and 20%, respectively). Moreover, at 2 and 4 hr after the administration (i.v.) of different doses of Eu1.6 (1, 4.98, and 49.8 μg/kg), Eu1.6 still displayed potent analgesic activity with the pain threshold increasing to 151 ± 11.1 g, 172 ± 43.8 g, 164 ± 36. and 139 ± 14.3 g, 152 ± 32.5 g, 153 ± 22.0 g (n = 8), respectively, from the starting values of 127 ± 13.8 g, 126 ± 20.5 g, 103 ± 9.5 g (n = 8) (Fig. 3c,d). The analgesic activity of Eu1.6 was further tested in the rat CCI pain model. The pain threshold significantly increased after the administration of different doses of Eu1.6 (Fig. 3e–h), and was similar to the results of PNL experiments. In the above PNL and CCI models, Eu1.6 exhibited higher analgesic activity than Vc1.1 and the combination of morphine and gabapentin, and the effects were sustained for longer duration.

### Eu1.6 inhibits nicotinic acetylcholine receptors (nAChR) with low potency

**Figure 4:**
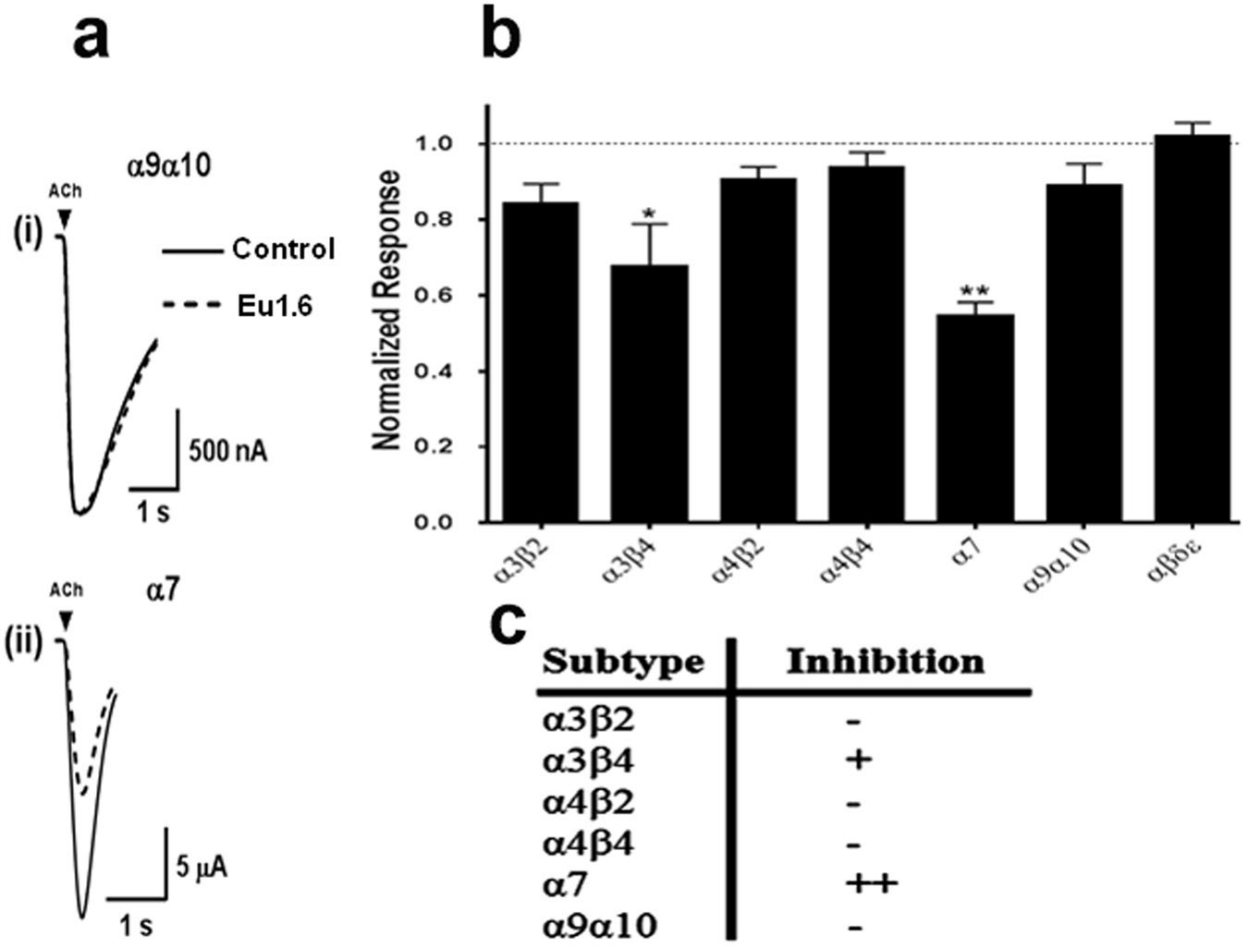
Eu1.6 inhibition of nicotinic acetylcholine receptor (nAChR) subtypes. Eu1.6 (10 µM) was applied by perfusion to oocytes expressing nAChRs as described in *Methods*. The error bars denote the S.E.M. of the data from four to six oocytes for each determination. (**a**) Representative ACh-evoked currents mediated by α9α10 and α7 nAChRs in the absence (control) and presence of 10 µM Eu1.6. (**b**) Bar graph of the mean ACh-evoked current amplitude of nAChR subtypes in the presence of 10 μM  Eu1.6. Data are presented as Mean ± SEM, Statistical significance was determined with One-way ANOVA, *p < 0.05, **p < 0.001. (**c**) A summary of the effect of Eu1.6 on various neuronal nAChR subtypes.

Given that Eu1.6 is an α-conopeptide, we tested Eu1.6 at various rat nAChR subtypes expressed in *Xenopus* oocytes. Eu1.6 (10 μM) inhibited the ACh-evoked current amplitude mediated by the rat neuronal α3β4 and α7 nAChR subtypes by 30 ± 5% and 45 ± 2% (n = 4–6), respectively (Fig. 4). At the same concentration, Eu1.6 inhibited the α9α10 nAChR subtype by only 11 ± 2% (n = 6) suggesting that the potent analgesic activity of Eu1.6 is unlikely to be due to the inhibition of α9α10 nAChRs.

### Eu1.6 inhibits HVA calcium currents in rodent DRG neurons

**Figure 5:**
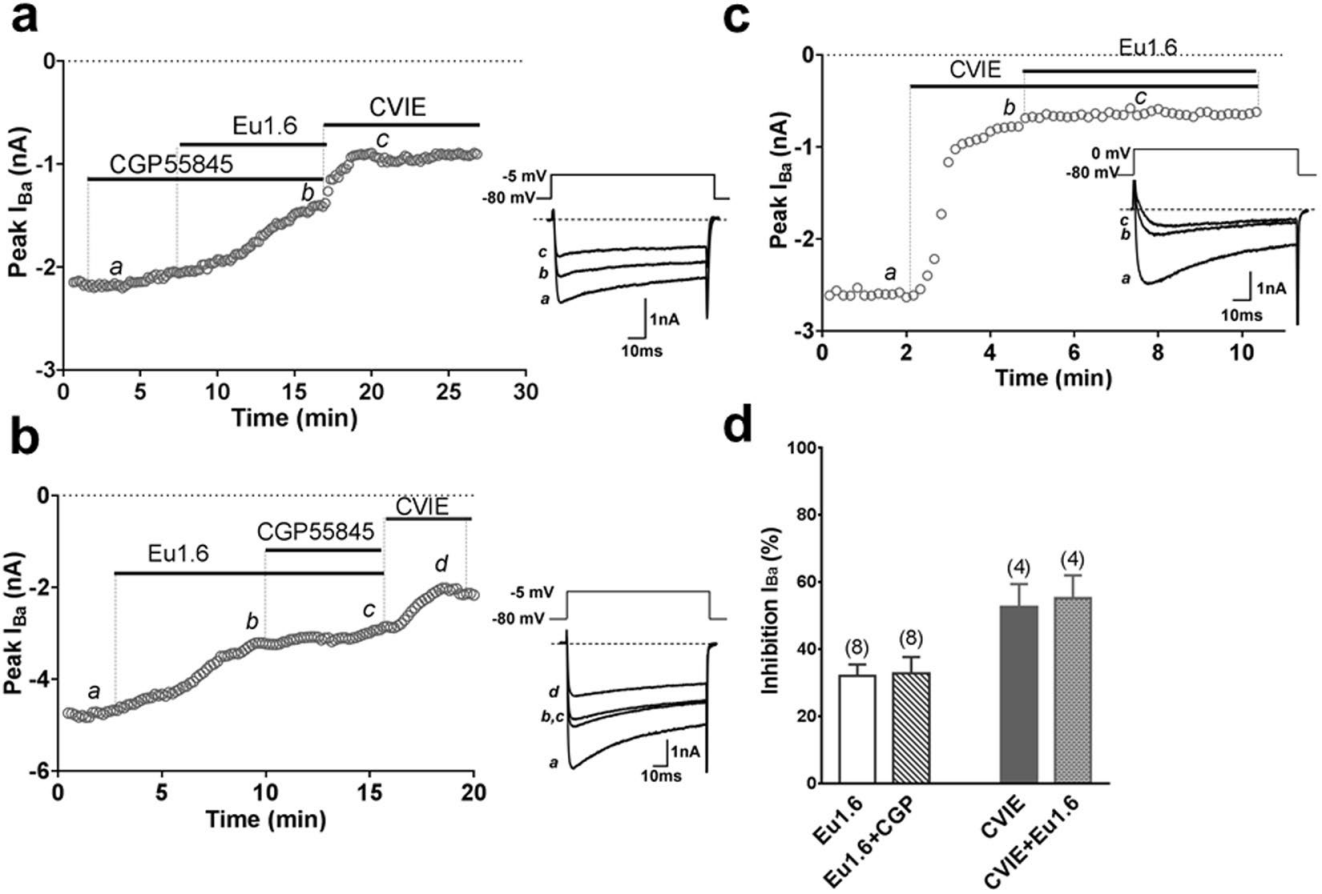
Eu1.6 inhibition of depolarization-activated Ba^2+^ currents is independent of GABA_B_ receptor activation in mouse DRG neurons. (**a**) Superimposed representative time plots of I_Ba_ inhibition by 1 µM Eu1.6 in the presence of 1 µM CGP55845in mouse DRG neurons. Bars indicate the bath application of CGP55845, Eu1.6 in the presence of CGP55845, and ω-conotoxin CVIE. Superimposed inward Ba^2+^ currents (*bottom insets*) obtained in the presence of 1 µM CGP 55845 (*a*), Eu1.6 in the presence of CGP 55845 (*b*), and 300 nM CVIE (*c*), are shown at the times indicated by lowercase letters. Dotted lines indicate zero-current level. (**b**) Time course of I_Ba_ inhibition by 1 µM Eu1.6 Eu1.6 in the presence of CGP55845 in mouse DRG neurons. Bars indicate the bath application of Eu1.6, Eu1.6 in the presence of CGP55845, and ω-conotoxin CVIE. Application of CGP 55845 did not antagonize the effect of Eu1.6 on HVA calcium channel. (**c**) Time course of the inhibition of I_Ba_ by 300 nM CVIE (*b*) and 1 µM Eu1.6 in the presence of 300 nM CVIE (*c*) in mouse DRG neurons. Inward I_Ba_ elicited by step depolarization applied at 0.1 Hz from a HP of −80 mV to −5 mV or 0 mV (as indicated in bottom inserts for each representative time course) plotted as a function of time. (**d**) Bar graph summary of inhibition of peak current amplitude by 1 µM Eu1.6, 1 µM Eu1.6 in the presence of 1 µM CGP55845, 300 nM CVIE, and 300 nM CVIE + 1 μM Eu1.6. Data are presented as Mean ± SEM. No significant difference was observed between inhibition of HVA calcium channel current by application of Eu1.6 and Eu1.6 + CGP55845 (*P* > 0.05, paired two-tailed t-test) and the inhibition of HVA calcium current by CVIE and CVIE + Eu1.6 (*P* > 0.05, paired two-tailed t-test). Number of experiments is indicated in parentheses.

A possible underlying mechanism of Eu1.6 action could involve the G protein-coupled GABA_B_ receptor-mediated inhibition of HVA calcium channels^33^. The 4/7 α-conopeptide, Vc1.1, and 4/3 α-conopeptide, Rg1A, inhibition of N-type calcium channel currents via GABA_B_ receptor activation was antagonized by the selective GABA_B_ receptor antagonists CGP55845 (1 µM) or phaclofen (50 µM). We tested 1 µM Eu1.6 in acutely dissociated mouse DRG neurons and observed inhibition of depolarization-activated Ca^2+^ channel current amplitude by 32.8 ± 4.9% (n = 8) in the absence and 32.1 ± 3.4% (n = 8) in the presence of CGP55845 (Fig. 5a,b). These results suggested that the inhibition of HVA calcium channels in DRG neurons by Eu1.6 was not mediated via GABA_B_ receptor activation (Fig. 5d). To determine which of the various HVA calcium channels in mouse DRG neurons are inhibited by Eu1.6, ω-conotoxin CVIE (1 µM) was applied to selectively block N-type calcium channels. No further inhibition of HVA calcium channel currents by Eu1.6 was observed in the presence of CVIE (Fig. 5c) suggesting that the N-type calcium channel is the primary target of Eu1.6 (Fig. 5d). Eu1.6 (10 µM) was also tested on voltage-gated sodium channels in DRG neurons and shown that Eu1.6 inhibited TTX-sensitive (TTX-S) and TTX-resistant (TTX-R) Na^+^ currents by 5.5 ± 2.1% and 16.5 ± 4.2% (n = 4–6), respectively. These results show that 10 µM Eu1.6 weakly inhibited TTX-R Na^+^ channels but was relatively inactive on TTX-S Na^+^ channels in rat DRG neurons (p < 0.05, t-test) (Supp. Fig. S4). Furthermore, Eu1.6 (1–10 µM) exhibited no activity at opioid receptors (Supp. Table S4), VR1 (vanilloid receptor subtype 1) or KCNQ1 channels expressed in HEK293 cells (Supp. Fig. S5c,d).

### Eu1.6 reversibly inhibits human Ca_V_2.2 channels expressed in HEK293 cells

**Figure 6:**
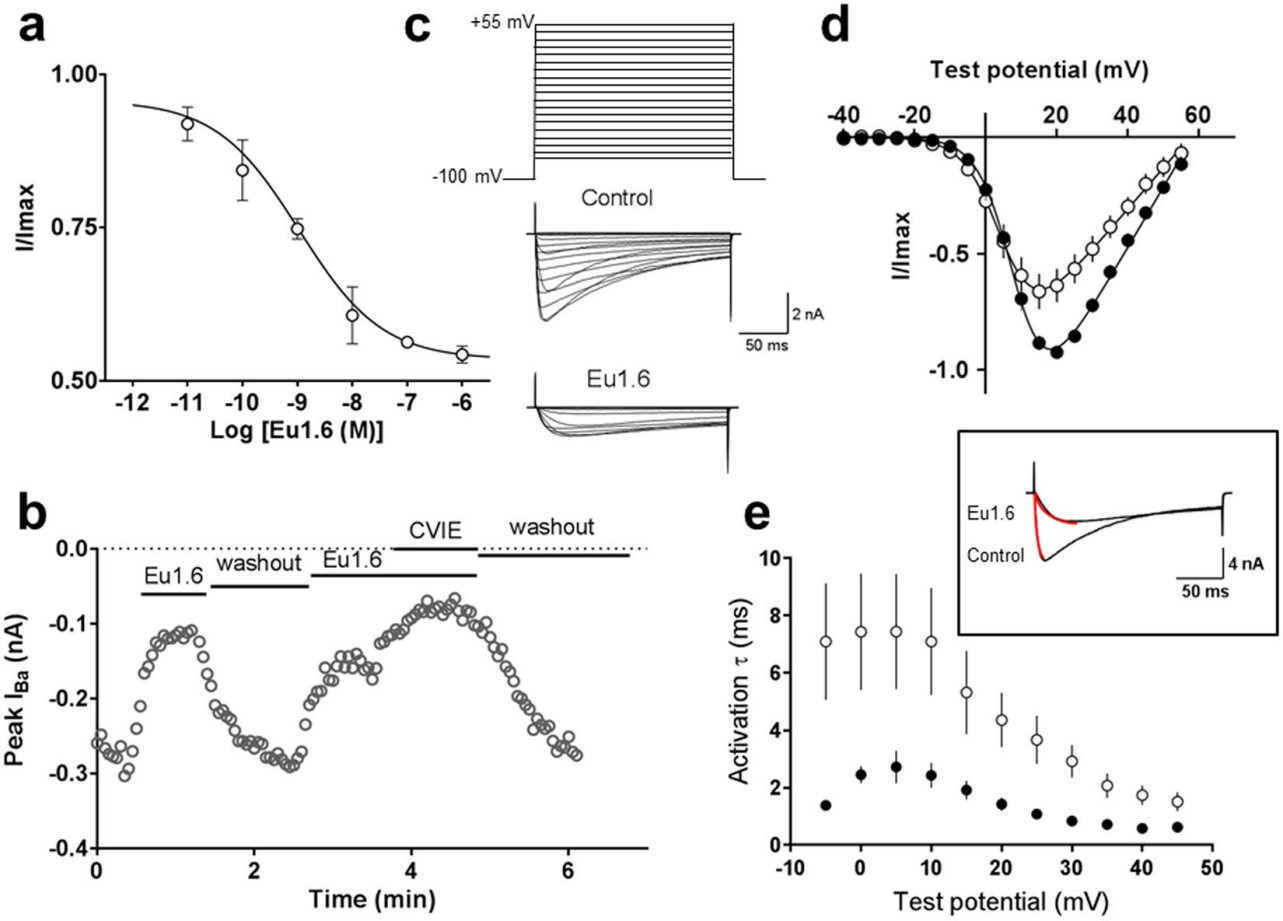
Eu1.6 concentration-dependent inhibition of human Ca_V_2.2 expressed in HEK293 cells. (**a**) Concentration- response relationship obtained for Eu1.6 inhibition of Ca_V_2.2 giving an IC_50_ of 1.1 nM and a maximum block of <50% (n = 4). (**b**) Ba^2+^current amplitude plotted as a function of time in the absence and presence of 1 µM Eu1.6 and 100 nM CVIE. Cells were activated by a depolarizing pulse from a holding potential of −80 mV to a test potential of +10 mv for 200 ms at 0.2 Hz, after reaching a plateau (max. block) cells were superfused (washout) with external bath solution for several minutes for complete recovery. (**c**) Voltage protocol used to determine the I-V relationship under control conditions and in the presence of 1 µM Eu1.6. Representative current traces recorded over a series of depolarizing testing potentials from a holding potential of −100 mV to −40 mV to +55 mV (∆5 mV increments) under control conditions and after >2 min. exposure to 1 µM Eu1.6 in the external bath solution. (**d**) I-V relationship recorded at certain time points in the absence (filled circle) and presence of Eu1.6 (open circle). (**e**) Activation kinetics (tau) measured in the absence (control, filled circle) and presence of 1 µM Eu1.6 (open circle) was determined by a mono-exponential function (inset) for a series of testing potentials derived from the same I-V traces.

To determine the mechanism of inhibition of HVA calcium channels by Eu1.6 observed in DRG neurons, we used HEK293 cells that stably expressed human Ca_V_2.2 channels composed of the α_1B_ subunit together with the β3 and α_2_δ1 subunits. Whole-cell currents recorded using 10 mM Ba^2+^ as the charge carrier were inhibited in a concentration-dependent manner with an IC_50_ of 1.1 nM (n = 4) (Fig. 6a) confirming that Eu1.6 directly inhibits Ca_V_2.2 channels. Ca_V_2.2 inhibition by 1 µM Eu1.6 was reversible upon washout and could be repeated in the same experiment (Fig. 6b). Eu1.6 (1–5 µM) did not inhibit either L- or T-type calcium channels expressed in HEK293 cells (n = 4–6) (Supp. Fig. S5a,b).

**Figure 7:**
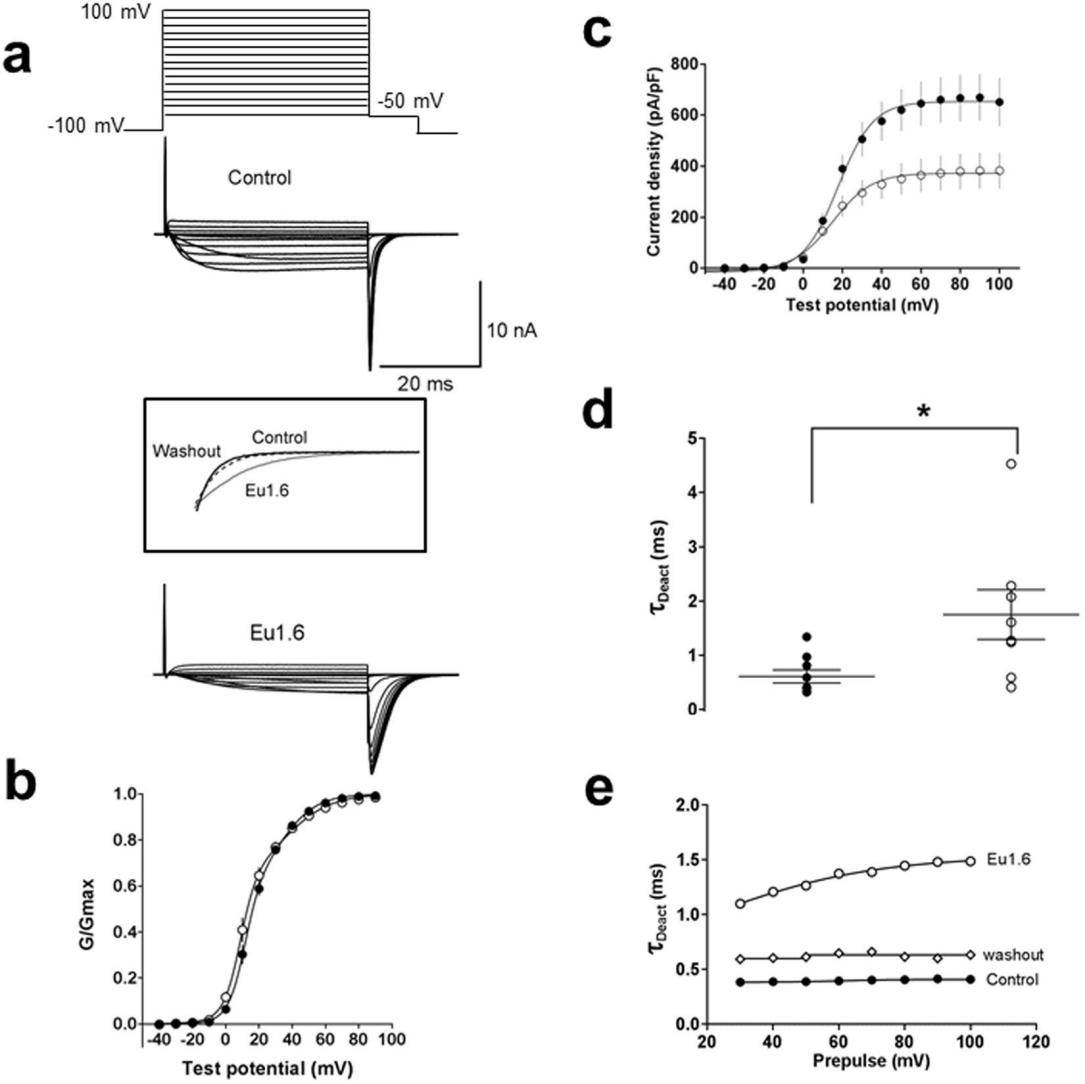
Eu1.6 slows deactivation kinetics of Ca_V_2.2 channel. (**a**) Tail currents were determined at a repolarizing potential (−50 mV) for 10 ms in the absence (control) and presence of Eu1.6. Insert: expanded time scale of superimposed tail currents obtained in the absence (control, black line; washout, dashed line) and presence of Eu1.6 (grey line). (**b**) Conductance-voltage (G-V) relationships determined from tail currents recorded in the absence (control, filled circle) and presence of Eu1.6 (open circle). The current was normalized to its maximum current and fitted with a double Boltzmann function. (**c**) Current density plot was derived from the current amplitude (pA) divided by the cell capacitance (pF). See Supp. Tables S2 and S3 for parameter and significance testing. (**d**) Deactivation kinetics (tau) was determined by a mono-exponential fit of tail currents. The scatter plot depicts the deactivation kinetics in the absence (filled circle) and presence of Eu1.6 (open circle) for a series of experiments at a single prepulse potential. Statistical significance was determined with an unpaired t-test, *p < 0.05. (**e**) Representative deactivation kinetics of currents traces recorded in the absence (control, filled circle), after incubation (>2 min.) with Eu1.6 (open circle) and upon washout (open diamond).

To investigate the effect of Eu1.6 inhibition on the Ca_V_2.2 current-voltage (I-V) relationship, several consecutive I-V curves were recorded and the I-V relationship obtained in the absence (control) and presence of Eu1.6 (1 µM) is shown in Fig. 6(c,d). Depolarization-activated Ba^2+^ current amplitude was inhibited by >30% over a wide range of membrane potentials in the presence of 1 µM Eu1.6 (Fig. 6d). Half-maximal activation (*V*_*0*.*5*_) and slope of the conductance-voltage relationship were not significantly shifted in the presence of Eu1.6 (*V*_*0*.*5*_ = 5.2 ± 1.9 mV, n = 14) compared with control (*V*_*0*.*5*_ = 8.7 ± 0.5 mV, n = 21) (p > 0.05, paired t-test, Supp. Table S2). The time to peak of the inward Ba^2+^ current fitted by a mono-exponential function (τ_act_) was determined at 0 mV (Fig. 6e) and τ_act_ significantly increased from 2.7 ± 0.9 ms (control) to 7.4 ± 0.5 ms (n = 6) in the presence of 1 µM Eu1.6 (p < 0.05, paired t-test, Supp. Table S2). The Ca_V_2.2 conductance-voltage (G-V) relationship was determined by measuring the repolarizing tail current amplitude from test potentials to −50 mV for 10 ms (Fig. 7a). The G-V curve was not significantly altered in the presence of Eu1.6 (Fig. 7b, Supp. Table S2), however, the peak current density was reduced by >40% in the presence of Eu1.6 (Fig. 7c) and time course of deactivation was significantly increased from 0.61 ± 0.12 ms to 1.75 ± 0.46 ms (n = 9; *p* < 0.05, paired t-test)) and reversible upon washout (Fig. 7d,e).

**Figure 8:**
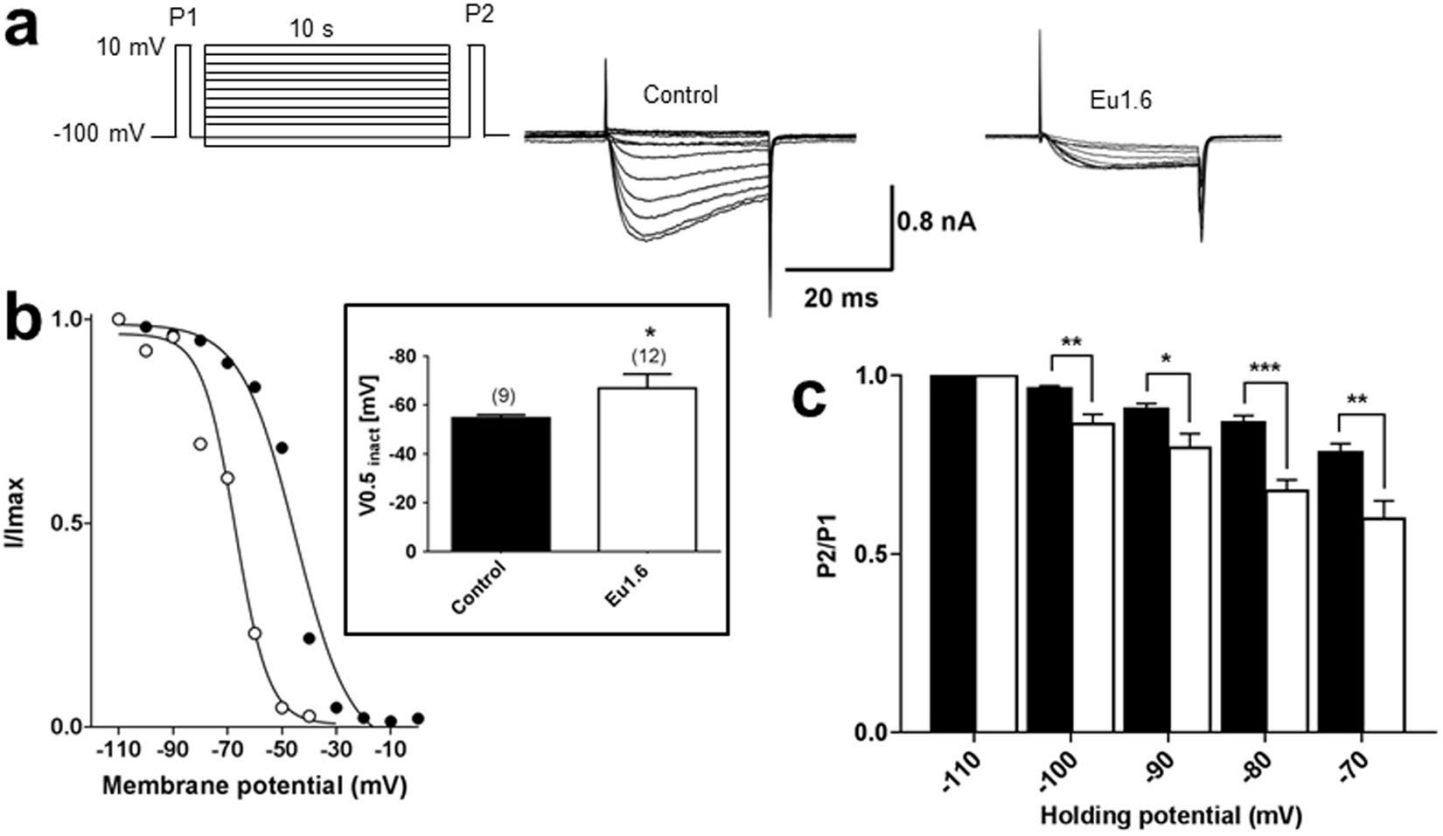
Eu1.6 shifts the voltage-dependence of steady-state inactivation (SSI) of the Ca_V_2.2 channel. The steady-state inactivation was determined by a triple pulse protocol. A long conditioning pulse over a series of membrane potentials (holding potentials), starting from −110 mV to 0 mV (10 mV increments, 10 s), was recorded immediately after a 50 ms prepulse to +10 mV (P1). Another brief pulse after the preconditioning pulse was recorded for 50 ms at +10 mV. (**a**) Representative current traces (P2) are shown in the absence and presence of 1 µM Eu1.6. Currents were normalized to the maximum current and plotted over a series of holding potentials. Data points were fitted with a single Boltzmann function as exemplified in (**b**). Insert: bar graph shows half-maximum inactivation (V_0.5,inact_) was shifted by more than 10 mV to more hyperpolarized potentials. (**c**) Bar graph of P2/P1 as a function of holding potential represents mean ± SEM values. Statistical significance for each holding potential was determined with an unpaired t-test, *p < 0.05, **p < 0.01 and ***p < 0.001.

To determine if Eu1.6 affected Ca_V_2.2 channel inactivation, the voltage-dependence of steady-state inactivation was investigated (Fig. 8a–c). Ca_V_2.2 steady-state inactivation was shifted by −12 mV to more hyperpolarized potentials from *V*_*0*.*5inact*_ of −54.7 ± 1.2 mV (control) to −66.8 ± 5.9 mV in the presence of Eu1.6 (n = 9; *p* < 0.05, unpaired t-test). Evaluation of Eu1.6 inhibition of Ca_V_2.2 at −110 mV compared with −70 mV revealed enhanced steady-state inactivation by Eu1.6 at hyperpolarized holding potentials (Fig. 8c) indicating that Eu1.6 preferentially inhibits the inactivated channel. This suggests that Eu1.6 is a potential novel state-dependent inhibitor of Ca_V_2.2 channels.

### Eu1.6 does not affect locomotor activity or the cardiovascular system

Conopeptides are known for their potential off-target effects due to systemic administration. To examine the possibility of any side effects of Eu1.6 on locomotion, activating or depressing effects of Eu1.6 on locomotor activity was tested by the rotarod test and spontaneous locomotor activity test. Furthermore, any potential effects of Eu1.6 on cardiac and respiratory function were assessed to evaluate the potential risk of a cardiac depressant effect. Globally, no physiological impairments of baseline parameters were observed with intramuscular injection of high doses (1–50 mg/kg) of Eu1.6 (Supp. Figs S1–S3).

## Discussion

According to the number of the amino acids between cysteines (-CCXnCXmC-), α-conopeptides are divided into several subtypes, such as 3/5, 4/3, 4/4, 4/6, 4/7 and 5/5 subtypes^12,34–36^. Until now, 4/3 and 4/7 α-conopeptide subtypes, such as Rg1A, Vc1.1, and PeIA, have been shown to exhibit analgesic activities because they target both α9α10 nAChRs and GABA_B_ receptors coupled to N-type calcium channels^19–21^. In contrast to the reported α- or αO-conopeptides^37^, Eu1.6 displayed only weak inhibitory activity at neuronal nAChR subtypes and did not activate GABA_B_ receptors to modulate N-type calcium channels. However, Eu1.6 did inhibit HVA, N-type calcium channel currents in isolated mouse DRG neurons whereby inhibition of HVA calcium channel currents by ω-conotoxin CVIE was not further inhibited by a maximally effective concentration of Eu1.6. This suggests that the Eu1.6 binding site(s) may overlap with that of ω-CVIE. Furthermore, Eu1.6 did not inhibit voltage-gated sodium channels in DRG neurons nor KCNQ1 channels or L- and T-type calcium channels expressed in HEK293 cells (Supp. Figs S4–S5).

In HEK293 cells expressing Ca_V_2.2 channels alone, Eu1.6 concentration-dependently inhibited depolarization-activated Ba^2+^ currents in a voltage- and state-dependent manner. Eu1.6 inhibition of human Ca_V_2.2 is reminiscent of a partial inhibitor with a maximal effect of 40% at 1 µM. The voltage-dependence of activation did not appear to be largely affected, however, the time course of activation was increased by >2.7-fold. In contrast, steady-state inactivation exhibited a statistically significant shift of approximately −12 mV which is consistent with that observed for the I-V relationship. Partial block of Cav2.2 may be explained by either an allosteric effect of the conopeptide on the channel or due to partial steric block of the pore^38^. However, single channel studies investigating unitary currents and open channel probability in the presence of Eu1.6 will be required to determine the precise mechanism of action of Eu1.6 block of Ca_V_2.2. To our knowledge, Eu1.6 is the first α-conotoxin reported to directly and reversibly inhibit N-type (Ca_V_2.2) calcium channels.

Protein alignment revealed that Eu1.6 shares lower sequence identity among α-conopeptides except Vc1.2 (75%). However, Vc1.2 potently inhibits α3β2, α7 and α9α10 nAChRs with IC_50_ values of 75 nM, 637 nM and 1000 nM, respectively^39^. The difference between Eu1.6 and Vc1.2 at target is likely derived from residues in the second loop because their amino acid residues in the first loop are similar.

Eu1.6 exhibits significant analgesic activity in neuropathic pain (PNL and CCI) models at a low dose (5.0~25.0 μg/kg) and was higher than Vc1.1 and the combination of morphine (5 mg/kg, sc) and gabapentin (100 mg/kg, i.g.). The latter combination is effective in current neuropathic pain therapy^40,41^. Similar results were observed in the CCI model. Interestingly, Eu1.6 was also highly effective after i.v. administration in PNL and CCI rats. This is first α-conopeptide to show potent analgesic activity for neuropathic pain via intravenous administration. Eu1.6 exhibits no apparent analgesic activity in sham rats, demonstrating its specificity for neuropathic pain. Importantly, at the high doses of 25 mg/kg (i.m.) and 100 mg/kg (i.v.), Eu1.6 did not exhibit any significant side-effects in acute tests of rats, and a more than 100-fold effective dose of Eu1.6 (5 mg/kg i.v.) had no significant effects on mice spontaneous locomotor activity (Fig. S2). Similarly, Eu1.6 doses of 166.3 μg/kg (i.m.) and 1 mg/kg (i.v.) did not show any significant effect on mice motor function, cardiac or respiratory function, demonstrating Eu1.6 has a high safety margin. In addition, Eu1.6 did not exhibit the morphine-like addictive symptoms after continuous administration of 24.90 µg/kg, 249 µg/kg, and 2490 µg/kg Eu1.6 for 8 days (Supp. Fig. S6).

In summary, we have characterized a novel α-conopeptide, Eu1.6, showing it to directly target N-type (Ca_v_2.2) calcium channels with high potency in contrast to its activity at neuronal nAChRs subtypes. Eu1.6 exhibited potent analgesic activity in the rat PNL and CCI neuropathic pain models at μg/kg levels after intramuscular (i.m.) and intravenous (i.v.) administration. Importantly, Eu1.6 did not exhibit any significant side-effects for spontaneous locomotor activity, cardiac and respiratory function and drug dependence. These findings suggest Eu1.6 is potent analgesic for neuropathic pain and is a novel lead for further drug design associated with pain treatment.

## Methods

### Cloning of Eu1.6

*Conus eburneus* were harvested near Hainan Island in the South China Sea. The venom ducts were dissected from living cone snails and frozen immediately in liquid nitrogen. Total RNA was extracted and purified from ~100 mg of homogenized venom duct tissue using TRIZOL Reagent (Invitrogen, Carlsbad, CA, USA) as described previously^42,43^.

1 µg of total venom duct RNA was used to generate cDNA using M-MLV reverse transcriptase with a 3′-RACE adaptor, and the resulting cDNA used as the template for the first 3′RACE PCR reaction. To amplify the cDNA encoding the novel α-conopeptides, the following nested primers pairs were employed. Forward primers, outer primer F1 (5′-ATG GGC ATG CGG ATG ATG TTC-3′) and inner primer F2 (5′-CTG TTG GTT GTC TTG GCA ACC AC-3′), were designed based on the conserved sequence of the signal peptides of known A-superfamily conotoxins. Reverse 3′-RACE outer primer R1 (5′-TAC CGT CGT CGT TCC ACT AGT GAT TT-3) and reverse 3′-RACE inner primer R2 (5′-CGC GGA TCC TCC ACT TGT GGT AGG G-3′), were provided in the 3′-Full RACE Core Set Ver.2.0 kit (TaKaRa Biotechnology, Dalian, China). Both the first and the second PCR amplification reactions included an initial denaturation step carried out at 94 °C for 4 min, followed by 30 amplification cycles (94 °C for 30 s, 56 °C for 30 s and 72 °C for 1 min) and a final extension step at 72 °C for 10 min. PCR products were analyzed on a 1% agarose gel, the appropriate bands purified, ligated into pGEM-T Easy Vectors and transformed into *E*. *coli*. DH5α cells (TIANGEN Biotech Co., Ltd., Beijing, China). Clones containing the desired inserts were identified by PCR and subsequently sequenced.

### Peptide synthesis, folding and disulfide connectivity analysis

The linear peptide Eu1.6 was synthesized by the solid-phase method using an ABI 433 A peptide synthesizer as described previously^44,45^. The peptide-resin was cleaved in a reagent mixture composed of 8.8 ml TFA, 0.5 g DTT, 0.5 ml H_2_O and 0.2 ml triisopropylsilane for 3 h at room temperature. The released peptide was filtered, precipitated with cold ether and folded in 0.1 M NH_4_HCO_3_ buffer (pH 8.2) at a concentration of 0.4 mg/ml for 24–48 h at room temperature. The folding procedure was monitored by HPLC. Following the oxidation step, the reaction was stopped by acidification with acetic acid to a pH of 4.0–5.0. The mixture was then filtered and directly loaded on to a 25 × 250 mm preparative C_18_ column using a preparative HPLC pump (Waters Delta Prep 4000). The column was washed with buffer A (0.1% TFA in H_2_O) at a flow rate of 2.5 ml/min and then with buffer B (0.1% TFA in acetonitrile) at a flow rate of 5 ml/min. The resulting peptide was further purified by semi-preparative RP-HPLC using a Kromasil 10.0 × 250 mm C18 column. Confirmation of the correct molecular mass was ascertained by mass spectrometry on a ProFLEXTM-III MALDI-TOF spectrometer.

The disulfide arrangement of Eu1.6 synthesized by the one-step oxidative folding was determined by comparison with peptide folding products of known disulfide connectivity as described previously^44–46^.

### NMR spectroscopy

Eu1.6 was dissolved in 500 μl of 20 mM phosphate buffer (pH 5.5) containing 10% D_2_O. NMR experiments, including TOCSY, NOESY and DQF-COSY, were conducted on a Bruker Avance 800 MHz spectrometer using a TXI cryoprobe. All spectra were collected at 303 °K. The mixing times of TOCSY were 30 and 70 ms, respectively. The mixing times used for NOESY experiments were 300, 400, 500 ms, respectively. H/D exchange experiment was performed at 303 °K by adding 500 μl D_2_O to the lyophilized sample.

Spectra were processed using NMRPipe software and peak analyses were conducted with the XEASY module in Cara (version 1.5.5)^47^. Spin systems were established by DQF-COSY and TOCSY spectra. NOE intensities in NOESY spectrum with a 500 ms mixing time were extracted by CARA 1.8.4.2 and converted into distance constraints. ^3^*J*_*Hα-NH*_ coupling constants were determined from DQF-COSY. A set of 100 structures was calculated using CYANA (version 2.1) with 239 non-redundant NOE and 8 dihedral angle constraints^44^. The final 20 conformers with the lowest energies were refined using Amber11. Disulfide bonds were checked by measuring the inter-nuclear distances in structures calculated without disulfide bond constraints and then added to the final calculation. 70.8% of the phi-psi torsion angles in the structure were in the most favored regions in the Ramachandran plot. The three-dimensional structure was displayed using the MOLMOL graphics program. The data, including chemical shifts, were submitted to the BMRB database with access codes 21060 for Eu1.6.

### Analgesic activity tests

Adult male Sprague-Dawley rats (SD, 220–250 g) (Beijing Animal Center, China) were housed in groups of eight and maintained on a 12 h light-dark cycle (light cycle from 8 am–8 pm) at a temperature of 23 ± 2 °C and a relative humidity of 50%. Food pellets and water were available *ad libitum*. All methods and experimental protocols were approved and carried out in accordance with the guidelines and regulations of the Beijing Institutes for Biological Sciences Animal Research Advisory Committee and conformed to the European Community directives for the care and use of laboratory animals.

Rat partial sciatic nerve injury (PNL) and chronic constriction injury (CCI) pain models were used to determine the analgesic activity of Eu1.6. The model was established as described previously^48,49^. In sham-operated rats, the same surgery was performed, but the nerve was not ligated. Rats scoring below 80 g or over 160 g before operation were rejected (20%). For analgesia measures, mechanical pressure application was stopped at 160 g. All behavioral testing was performed seven days after surgery. Successful PNL and CCI models were defined as a 30–50% decrease in mechanical paw withdrawal threshold (PWT) for the ipsilateral hind paw. PNL and CCI rats were subjected to mechanical stimulation using the paw pressure test to assess hyperalgesia-like responses in the hind paw ipsilateral to the ligated nerve. Nociceptive thresholds, expressed in grams, were measured with Ugo Basile analgesiometer (Ugo Basile, Italy) to produce increasing pressure to the hind paw until paw withdrawal occurred. The effects of drugs were systematically compared on the paw withdrawal to pressure. Paw withdrawal thresholds were measured at 2, 4 and 6 h following intramuscular (i.m.) and intravenous (i.v.) administration. Data are expressed as mean paw withdrawal threshold (g).

Seven days after surgery, successful rat models were randomly assigned to six groups that each received one of the following treatments: sterile saline (negative control), Vc1.1, a combination of morphine (5 mg/kg) and gabapentin (100 mg/kg) (positive control) or Eu1.6. Eu1.6 was dissolved in 0.9% sterile saline to a volume of 200 μl and administered by intramuscular injection (0.5 μg/kg (0.3 nmol/kg), 2.49 μg/kg (1.5 nmol/kg) or 24.9 μg/kg (15 nmol/kg) close to the injury site in the mid-thigh region or by intravenous injection (1 μg/kg (1 nmol/kg), 4.98 μg/kg (3.0 nmol/kg) or 49.8 μg/kg (30 nmol/kg)). α-Conotoxin Vc1.1 was administered (27.2 μg/kg, 15 nmol/kg) intramuscularly morphine by subcutaneous injection (sc) and gabapentin by intragastric administration (i.g.).

### Two-electrode voltage clamp experiments in oocytes

Oocyte electrophysiology was carried out as described previously^44,46^. Rat nAChR subunits α2, α3, α4, α7, β2 and β4 were in pNKS2 vector, α9 in pGEMHE vector, α10 in pSGEM vector and human α7 in pMXT. *Xenopus laevis* oocytes were injected with 5 ng of cRNA, and then kept at 18 °C and maintained in sterile ND96 buffer (96 mM NaCl, 2 mM KCl, 1.8 mM CaCl_2_, 1 mM MgCl_2_, and 5 mM HEPES, at pH 7.4) supplemented with 50 mg/l gentamycin and 100 μg/IU per milliliter penicillin/streptomycin for 2–5 days before recording. All methods and experimental protocols were approved and carried out in accordance with the guidelines and regulations of the Beijing Institutes for Biological Sciences Animal Research Advisory Committee and  RMIT University, Melbourne, Australia. Membrane currents from *Xenopus* oocytes were recorded using a two-electrode voltage clamp setup (virtual ground circuit) with either an Axoclamp 900A or GeneClamp 500B amplifier (Molecular Devices, Sunnyvale, CA). All recordings were made at room temperature (21–23 °C) using a bath solution of ND96 as described above, and oocytes were voltage clamped at a holding potential of –80 mV. Voltage-recording and current-injecting electrodes were pulled from borosilicate glass (GC150T-7.5; Harvard Apparatus Ltd., Holliston, MA) and had resistances of 0.3–1.5 MΩ when filled with 3 M KCl. During recordings, the oocytes were perfused continuously at a rate of 2 ml/min including application of ACh (200 μM for α7 and 50 μM for all other nAChR subtypes) for 2 sec and 180–240 sec washout periods between applications. No desensitization or run down of currents were observed between controls. Oocytes were incubated with the peptide for 300 sec before application of ACh plus peptide. Data were sampled at 500 Hz and filtered at 100 Hz. Peak ACh-evoked current amplitude was recorded using pClamp 10 software (Molecular Devices). The effects of peptide on ACh-evoked currents were defined as peak current amplitudes relative to the average peak current amplitude of 3–5 control ACh applications recorded before pre-incubation with the peptide. All electrophysiological data were presented as the mean  ± SEM of measurements taken from 4–6 oocytes for each nAChR subtype. Significance of inhibition was calculated by One-way ANOVA analysis compared to 0% inhibition (represented by value 1.0 in Fig. 4b) using GraphPad Prism (GraphPad Software Inc., La Jolla, CA).

### Whole-cell electrophysiological recordings from rodent DRG neurons

The protocol of dorsal root ganglion (DRG) neuron preparation was approved and carried out in accordance with the University of Wollongong and RMIT University Animal Ethics Committees guidelines and regulations. Briefly, mice were euthanized with isoflurane inhalation following decapitation. DRG neurons were collected, digested, and mechanically triturated from ganglia of 8–10 week-old C57BL/6 mice as described previously^19^. Cell suspensions were washed twice in supplemented DMEM (containing 10% heat-inactivated FBS and 1% penicillin/streptomycin) (Thermo Fisher Scientific), resuspended in supplemented DMEM and plated on poly-D-lysine/laminin-coated 12 mm round coverslips (BD Biosciences, Bedford, MA, USA), incubated at 37 °C in high relative humidity (95%) and controlled CO_2_ level (5%), and used within 16–36 h. In rodent DRG neurons, membrane currents through HVA calcium channels were recorded in the whole-cell configuration of the patch clamp technique with an Axopatch 200B amplifier (Molecular Devices, LLC, Sunnyvale, CA) at room temperature (22–24 °C). Extracellular (bath) solution containing (in mM): 150 tetraethylammoonium (TEA)-Cl, 2 BaCl_2_, 10 D-glucose and 10 HEPES, pH 7.4. Fire-polished borosilicate (C150TF-7.5, Harvard Apparatus Ltd.) patch pipettes with tip resistance values of 1.5–2.2 MΩ were filled with an intracellular solution containing (in mM): 140 CsCl, 1 MgCl_2_, 5 Mg-ATP, 0.1 Na-GTP, 5 1,2- bis(O-aminophenoxy)ethane-N,N,N′,N′ -tetraacetic acid tetracesium salt (BAPTA)-Cs_4_, and 10 HEPES-CsOH, pH 7.3. A voltage protocol using step depolarization from −80 mV to −5 or 0 mv was used when examining HVA calcium channel current. Currents were generated by a computer using pCLAMP 10 software (Molecular Devices, LLC), filtered at 3 kHz and sampled at 10 kHz. Leak and capacitive currents were subtracted using a −P/4 pulse protocol.

### Whole-cell calcium channel current recordings from HEK293 cells

Whole-cell Ba^2+^ currents of the N-type human isoform *α*_1B_ (37b), β3 and α_2_δ1 were recorded from stable transfected HEK293 cells. External bath solution contained (in mM): 90 NaCl, 10 BaCl_2_, 30 TEA-Cl, 1 MgCl_2_, 5 CsCl, 10 D-glucose, 10 HEPES, pH adjusted to 7.3 with TEA-OH. Borosilicate fire polished pipettes (2–3 MΩ) were backfilled with internal solution containing (in mM): 120 K-gluconate, 2 MgCl_2_, 5 EGTA, 5 NaCl, 4 Mg-ATP, 10 HEPES, pH adjusted to 7.2 with CsOH. The series resistance (usually <8 MΩ) and the cell capacitance measured with the MultiClamp 700B Amplifier (Molecular Devices, LLC) and compensated by ~70% avoiding current oscillation. Ba^2+^ currents were sampled at 10 kHz and low pass-filtered at 1 kHz. Raw data acquisition was obtained with pClamp 10 and stored to a common CPU. Data analysis and curve fitting were performed by using GraphPad Prism software (San Diego, CA). The current-voltage (I-V) relationship was obtained from a holding potential (HP) of −100 mV and pulsed for 200 ms to a series of test potentials (TP) starting from −40 mV to +50 mV (Δ10 mV increments). The I-V relationship was fitted with the equation: *I*_*Ba*_^*2+*^  = *G*_max_*(*V* − *E*_*rev*_)*/*(*1* + *exp* ((*V* − *V*_*0*.*5*_)*/k*)) where *G*_*max*_ is the maximum conductance: *E*_*rev*_ is the reverse potential; *V*_*1/2*_ is the half-maximal activation potential and *k* is the slope. The steady-state inactivation was obtained from a HP of −100 mV stepping to a normalizing pulse of +10 mV for 50 ms followed by a series of 10 sec conditioning pulses from −110 mV to +10 mV and a 50 ms test pulse to +10 mV. Data were fitted with a Boltzmann equation: *I*_*relative*_ = *I*_*min*_ + (*I*_*max*_ − *I*_*min*_)*/*(*1* + *exp* ((*V*_*0*.*5*_* − V*)*/k*) where *I*_*relative*_ is the normalized current; *I*_*min*_ and *I*_*max*_ are the current extremes and *V*_*0*.*5*_ is the half-maximal inactivation. Activation kinetics was determined by tail currents where cells were depolarized by a 20 ms test pulse of an activating potentials starting from −50 mV to +100 mV. Tail currents were then measured after repolarization to −50 mV for 10 ms and normalized and fitted with a dual Boltzmann respectively, *G/G*_*max*_ = *F*_*low*_*/*(*1* + *exp* ((*V*_*0*.*5 low*_ − *V*)*/k*_*low*_))*/*(*1 F*_*low*_)*/*(*1* − *exp*((*V*_*0*.*5 high*_ − *V*)*/k*_*high*_), *w*ith *G* as the tail current and *G*_*max*_ as the peak tail current, *F*_*low*_ is the fraction of the low-threshold component;*V*_*0*.*5 low*_, *V*_*0*.*5 high*_, *k*_*low*_, and *k*_*high*_ are the half-activation potentials and slope factors for the low and high threshold components and *V*_*0*.*5 act*_ was determined when *G* = 0.5*G*_*max*_. Whole-cell currents were leak and capacity corrected with an online −*P/4* protocol. Eu1.6 was dissolved in deionized water and stored at stock solution of 10 mM. Cells were superfused by a gravity driven system. All experiments were carried out at room temperature (21–23 °C).

### Data analyses

The results of behavioural testing were expressed as mean ± SEM or SD and analyzed by separate one-way analysis of variance (ANOVA) followed by the Student–Newman–Keuls method for multiple comparison tests at the 0.05 level of significance.

## Electronic supplementary material

Supplementary Information
