# Supplementary material for: A novel α-conopeptide Eu1.6 inhibits N-type (CaV2.2) calcium channels and exhibits potent analgesic activity
Source: Sci Rep. 2018 Jan 17;8:1004. doi: 10.1038/s41598-017-18479-4 (PMC5772529; doi:10.1038/s41598-017-18479-4)
Supplement: Supplementary file 1 — Supplementary Information [file 41598_2017_18479_MOESM1_ESM.pdf]

Supplementary Information

A novel α-conopeptide Eu1.6 inhibits N-type ($Ca_V2.2$) calcium channels and exhibits potent analgesic activity

Zhuguo Liu[1,†], Peter Bartels[2,†], Mahsa Sadeghi[2,3,†] Tianpeng Du[4], Qing Dai[1], Cui Zhu[1], Shuo Yu[1], Shuo Wang[1], Mingxin Dong[1], Ting Sun[1], Jiabin Guo[5], Shuangqing Peng[5], Ling Jiang[4,*], David J. Adams[2,3, *] and Qiuyun Dai[1,*]

[1] Beijing Institute of Biotechnology, Beijing 100071, China;
[2] Health Innovations Research Institute, RMIT University, Melbourne, VIC 3083, Australia;
[3] Illawarra Health and Medical Research Institute (IHMRI), University of Wollongong, Wollongong, NSW 2522, Australia;
[4] Key Laboratory of Magnetic Resonance in Biological Systems, National Center for Magnetic Resonance in Wuhan, State Key Laboratory of Magnetic Resonance and Atomic and Molecular Physics, Wuhan Institute of Physics and Mathematics, Chinese Academy of Science, Wuhan 430071, China;
[5] Institute for Disease control and Prevention, Beijing 100071, China;

M. S. and D. J. A. current address: Illawarra Health and Medical Research Institute (IHMRI), University of Wollongong, Wollongong, NSW 2522, Australia,

[†] These authors contributed equally to this work.

[*]Correspondence to: djadams@uow.edu.au; qy_dai@yahoo.com; lingjiang@wipm.ac.cn.

Key words: α-conopeptides, N-type calcium channels, voltage- and state- dependent inhibition, $GABA_B$ receptors, pain, analgesics.

Supplementary Methods

Rotarod test. Motor coordination and balance were tested as described previously[1,2]. Three days before the test, animals were trained at a speed of 10 rpm for 10 min once a day. Kuming mice (20 ± 2 g) were placed on an accelerating rotarod treadmill (YLS-4C, Shandong Academy of Medical Sciences, China). The peptides (16.6, 83.2 and 166.3 μg/kg) or saline were administered intramuscularly (i.m.) to the mice (n = 8) in a volume of 20 μl. After 120 min, the mice were placed on the rotating and the rod was accelerated from 0 to 30 rpm over a 5 min interval. The time during which the mice remained balanced on the rod was recorded (5 min as the maximum time). All methods and experimental protocols were approved and carried out in accordance with the guidelines and regulations of the Beijing Institutes for Biological Sciences Animal Research Advisory Committee and conformed to the European Community directives for the care and use of laboratory animals.

Spontaneous locomotor activity. Kunming mice (16 ± 2 g) were randomly divided into several dose groups and a normal saline (NS) control group with 10 animals in each group, half females and half males. Horizontal locomotor activity was recorded in boxes (25 ×25 × 35 cm) using JLBehv-LAG-4 animal behavior analyzer (Shanghai Jiliang Software Technology Co. Ltd., China). Animals were administrated intravenously with Eu1.6 freeze-dry powder (Eu1.6 freeze-dry powder consisted of 2 % Eu1.6, 1% methionine and 97% mannitol) at 0, 5, 50, and 250 mg/kg dissolved in saline. 2, 8 and 24 h after the administration (i.v.) of Eu1.6 or saline, each mouse was put into an individual box and adapted to the environment for 2 min, the total moving distance and time in 6 min were then recorded.

Drug dependence. Kunming mice (20 ± 2 g) were randomly divided into several dose groups, morphine and a NS control group with 10 animals in each group. Mice were injected every 12 h with Eu1.6 (30, 50, 100 nmol/kg, 100 μl, i.m.), morphine (100 mg/kg, 100 μl, s.c) and saline (100 μl, i.m.) at 08:30 and 20:30 for 6 days, respectively. On the day seven, the abstinence syndrome was precipitated by administering an i.p. injection of naloxone (5.0 mg/kg, 400 μl) 6 h after administration of same doses of

peptides, morphine and saline. Each mouse was immediately placed in a square observation box (30 cm x 30 cm x 50 cm) and the number of jumps was recorded over a 15 min period.

Cardiac and respiratory function test. 24 Beagle dogs were randomly divided into four groups (3 female/3 male per group). Animals were administrated intravenously with Eu1.6 freeze-dry powder (Eu1.6 freeze-dry powder consisted of 2 % Eu1.6, 1% methionine and 97% mannitol) at 1, 10, and 50 mg/kg dissolved in saline (1 ml/kg) or the vehicle solution (1 ml/kg). Animals were placed on a warming pad maintaining at 37 ºC and anesthetized by 1-2% isoflurane inhalation. Surface ECG (QRS, PRQ, and QT) was continuously monitored by inserting subcutaneous needle electrodes in a limb lead II electrograph. Cardio-haemodynamic parameters (heart rate, aortic blood pressure, systolic blood pressure, and mean blood pressure) were measured as described previously[3]. QTc were calculated using the Fridercia's rate equation as QT/RR0.33.A respiratory belt was placed at the position of each animal to record respiratory rate. All parameters were measured using MP150 data acquisition and analysis system (BIOPAC Systems Inc., CA, USA) 30 min before and 15 min, 30 min, 45 min, 1 h, 1.5 h, 2.0 h, 2.5 h, and 3.0 h after Eu1.6 or vehicle treatment. Animals were monitored during 24 h after drug treatment. All animal experiments were performed according to a protocol (No. 2013-010) approved by the Institutional Animal Care and Use Committee in compliance with the Guide for the Care and Use of Laboratory Animals published by the US National Institutes of Health.

Opioid receptor binding assay. Ligand binding experiments were carried out with [^{3}H]diprenorphine for opioid receptors as described previously[4,5]. Competition inhibition by peptides and morphine of [^{3}H]diprenorphine binding to opioid receptors was performed in the absence or presence of various concentrations of each drug. Binding was carried out in 50 mM Tris.HCl buffer (pH7.4) at 37℃ for 30 min in triple in a final volume of 0.5 ml with 30 μg of membrane protein prepared from CHO cell expressing human κ-, rat μ- or rat δ-opioid receptors. Naloxone (10 μM) was used to define nonspecific binding. Bound and free [^{3}H]diprenorphine were separated by filtration under reduced pressure with GF/B filters. Radioactivity on filters was

determined by liquid scintillation counting.

Electrophysiological recording of TTX-S and TTX-R Na^+ currents in DRG neurons. Whole-cell voltage clamp recordings of voltage-gated ionic currents were made in rat DRG neurons which were acutely dissociated from 30-day old Sprague–Dawley rats and maintained in short-term primary culture according to the method described by Xiao and Liang[6]. All methods and experimental protocols were approved and carried out in accordance with the guidelines and regulations of the Beijing Institutes for Biological Sciences Animal Research Advisory Committee. DRG neurons with large diameter (>35 μm) and those with relatively small diameter (<20 μm) were chosen for measuring TTX-S and TTX-R Na^+ currents, respectively. TTX (final concentration at 200 nM) was used to isolate TTX-R Na^+ currents from TTX-S Na^+ currents. Electrophysiological recording was performed as described previously[7]. TTX-S and TTX-R Na^+ currents were evoked by a 50 ms depolarizing step to −10 mV and +10 mV, respectively, from a holding potential of −80 mV.

Electrophysiological recording of L- and T-type calcium channels, KCNQ1 and VR1 expressed in HEK293 cells. Human embryonic kidney (HEK) 293 cells were cultured in modified Eagle's medium (DMEM, Gibco) supplemented with 10% fetal bovine serum (FBS, Gibco) 100 units of penicillin, 100 units of streptomycin at 37 ℃ incubator with 5% CO_2. Cells were transferred to a 24-well plate before transfection, and then transfected with the cDNA (Cav1.2(α_{1C}), Cav3.2(α_{1H}), VR1, KCNQ1) for 4 h and the transiently transfected using lipofectamine2000 (Invitrogen) according to the manufacturer's protocol.

Whole-cell voltage clamp recordings were performed ~24 h after transfection as described previously[8,9]. The patch pipettes were pulled from borosilicate glass capillaries with resistance of 2–4 MΩ in HEK293 cell experiments when filled with pipette solution. The extracellular solution for calcium channel experiments contained (in mM): 135 N-methyl-D-glucamine (NMDG), 20 $BaCl_2 \cdot 2\ H_2O$, 2 $MgCl_2 \cdot 6\ H_2O$, and 10 HEPES, pH ~7.4. Intracellular solution contained (in mM): 135 CsCl, 10 NaCl, 10 HEPES, 5 EGTA, and pH was adjusted to 7.2 with CsOH. The extracellular contained (in mM) in KCNQ1 channel and VR1 channel experiments: 140 NaCl, 5 KC1, 1

$MgC1_2{\cdot}6H_2O$, 10 HEPES, 10 D-glucose, pH ~7.4 with NaOH. The intracellular solution was the same as for calcium channel experiments. Membrane currents were recorded using a PC2C patch clamp amplifier with its corresponding software (InBio LifeScience Instrument Co., Ltd, China) and the currents were typically digitized at 100 kHz. Compensation of series resistance was 85% and ionic currents were filtered at 5 kHz. All experiments were performed at room temperature (20–24 °C).

Data analyses. The results of behavioural testing experiments were expressed as mean ± SEM or SD where stated, and analysed by separate one-way analysis of variance (ANOVA) followed by the Student–Newman–Keuls test for multiple comparison tests at the 0.05 level of significance. The data of spontaneous locomotor activity test and cardiac and respiratory function test were analyzed by two tailed test, Levene's test and t-test.

Supplementary Results

Supplementary Table S1. Structural statistics of the ensemble of 20 structures of Eu1.6 after CYANA calculation.

Parameters	Values [a]
Target function	0.53
NOE distance constraints	239
Intraresidue	136
Sequential	58
Medium range (1<|i-j|<5)	41
Long range (|i-j|≧5)	4
Dihedral angle	8
NMR constraint violations	
Number of NOE violations ≧ 0.2Å	0
Number of dihedral angle violations ≧ 5	0
RMSD deviation from the mean structure(Å)	
Backbone atoms	0.18 ± 0.05
Heavy atoms	0.44 ± 0.11
Ramachandran statistics analyzed using PROCHECK-NMR	
Residues in most favored regions	70.8%
Residues in allowed regions	29.2%
Residues in disallowed regions	0

[a] Values taken before energy minimization using Amber force field

Supplementary Table S2. Effect of Eu1.6 (1 μM) on human $Ca_V2.2$ channel current-voltage relationship (*I-V*) and voltage-dependence of activation (*G-V*).

	I-V		***G-V***					
	$V_{0.5}$ **(mV)**	***k***	$V_{0.5\ act./low}$ **(mV)**	k_{low}	F_{low}	$V_{0.5\ act./high}$**(mV)**	k_{high}	F_{high}
**Control**	8.7 ± 0.5	–4.8 ± 0.3	11.5 ± 1.5	5.0 ± 1.8	0.6 ± 0.3	33.1 ± 12.8	11.1 ± 2.6 (13)	0.4 ± 0.3
**Eu1.6**	5.2 ± 1.1	–5.2 ± 0.7	8.7 ± 1.0	4.9 ±1.4	0.6 ± 0.2	35.0 ± 11.3	13.9 ± 2.9 (9)	0.4 ± 0.2

$V_{0.5\ act./low}$ and $V_{0.5\ act./hig}$ are the half-maximal activation potential of low- and high-threshold components, respectively. ***k*** is the slope factor, F_{low} and F_{high} are the fraction of low- and high-threshold component, respectively.

Supplementary Table S3. Steady-state inactivation (*SSI*) and normalized current amplitude as a function of holding potential of human $Ca_V2.2$ in the absence (control) and presence of Eu1.6 (1 μM).

	$V_{0.5\ inact.}$**(mV)**	**k**	**–100 mV**	**–90 mV**	**–80 mV**	**–70 mV**
**Control**	-54.7 ± 1.2	10.7 ± 1.1 (9)	0.96 ± 0.01 (9)	0.90 ± 0.02 (7)	0.87 ± 0.02 (9)	0.78 ± 0.03 (9)
**Eu1.6**	-66.8 ± 5.9*	21.4 ± 7.6 (12)	0.86 ± 0.03 (10)**	0.80 ± 0.04 (8)*	0.68 ± 0.03 (10)***	0.60 ± 0.05 (10)**

* $p < 0.05$, ** $p < 0.01$ and *** $p < 0.001$ (unpaired t-test), $V_{0.5\ inact.}$ is the half-maximal inactivation potential and ***k*** is the slope factor. Number of observations is given in parentheses. Normalized calcium channel current amplitude obtained at different holding potentials with respect to the current amplitude obtained at a holding potential of –110 mV.

Supplementary Table S4. Displace [^{3}H]diprenorphine binding to opioid receptors by Eu1.6.

Opioid receptor	μ-receptor		κ-receptor		δ-receptor	
Eu1.6 (μM)	10	1	10	1	10	1
Inhibition (%)	4.4 ± 0.1	0.0	4.9 ± 0.1	0.0	7.7 ± 0.1	0.0

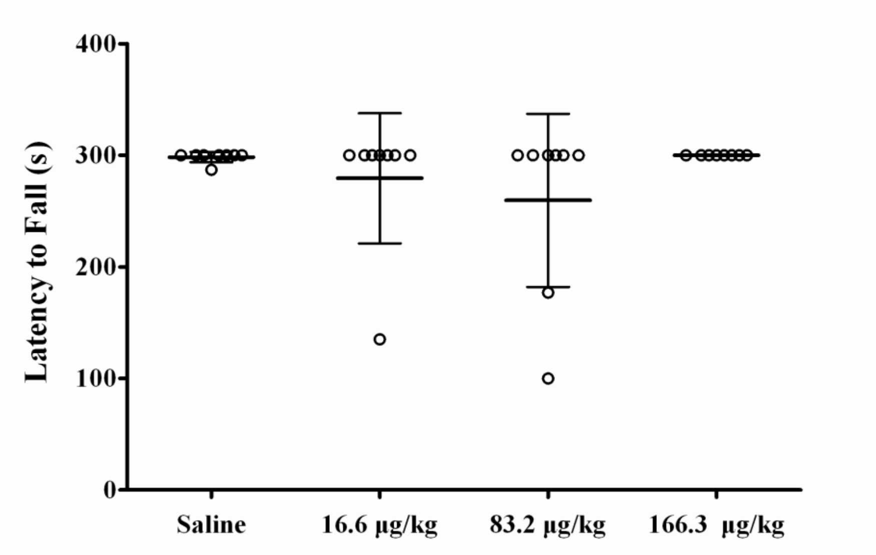

Supplementary Figure S1. Eu1.6 has no significant effects on coordinated locomotion in mice. 120 min after the administration (i.m.) of 16.6, 83.2, and 166.3 μg/kg of Eu1.6, the time spent on the rotarod is 298.4 ± 4.6 s, 279.4 ± 58.3 s, 259.6 ± 77.5 s and 300.0 ± 0.0 s, respectively. All peptide groups were not significantly different from saline (n = 8 per group, $p > 0.05$, t-test). Data represent the mean ± SD.

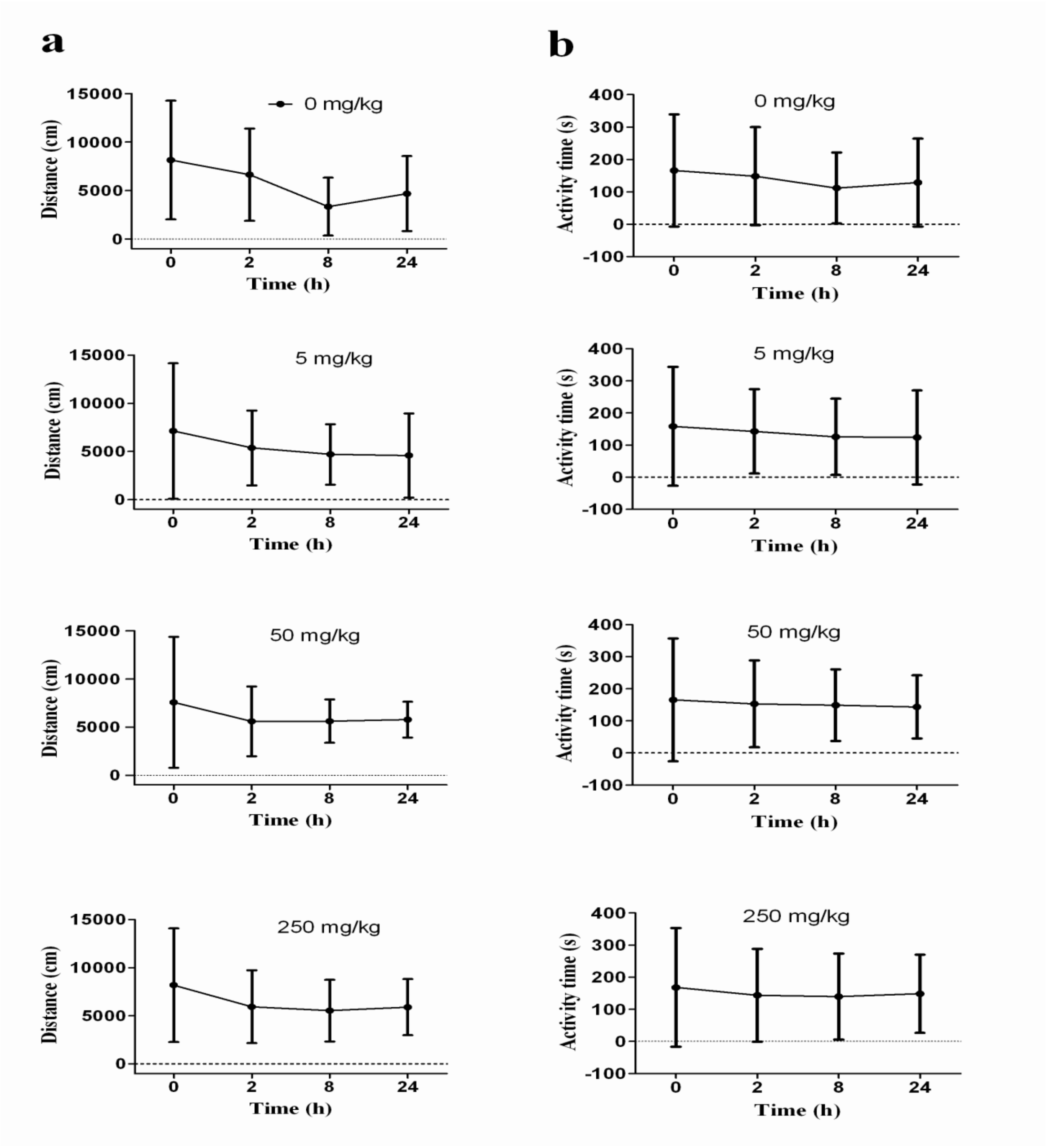

Supplementary Figure S2. Eu1.6 had no significant effects on spontaneous locomotor activity (SLA) in mice. (A) Moving distance (LHS column) and activity time (RHS column) of SLA of mice (n = 10) in 6 min, 0, 2, 8 and 24 h after administration (i.v.) of Eu1.6 (0, 5, 50 and 250 mg/kg). All peptide groups were not significantly different from vehicle (p > 0.05) at same time point. Data represent the mean ± SD.

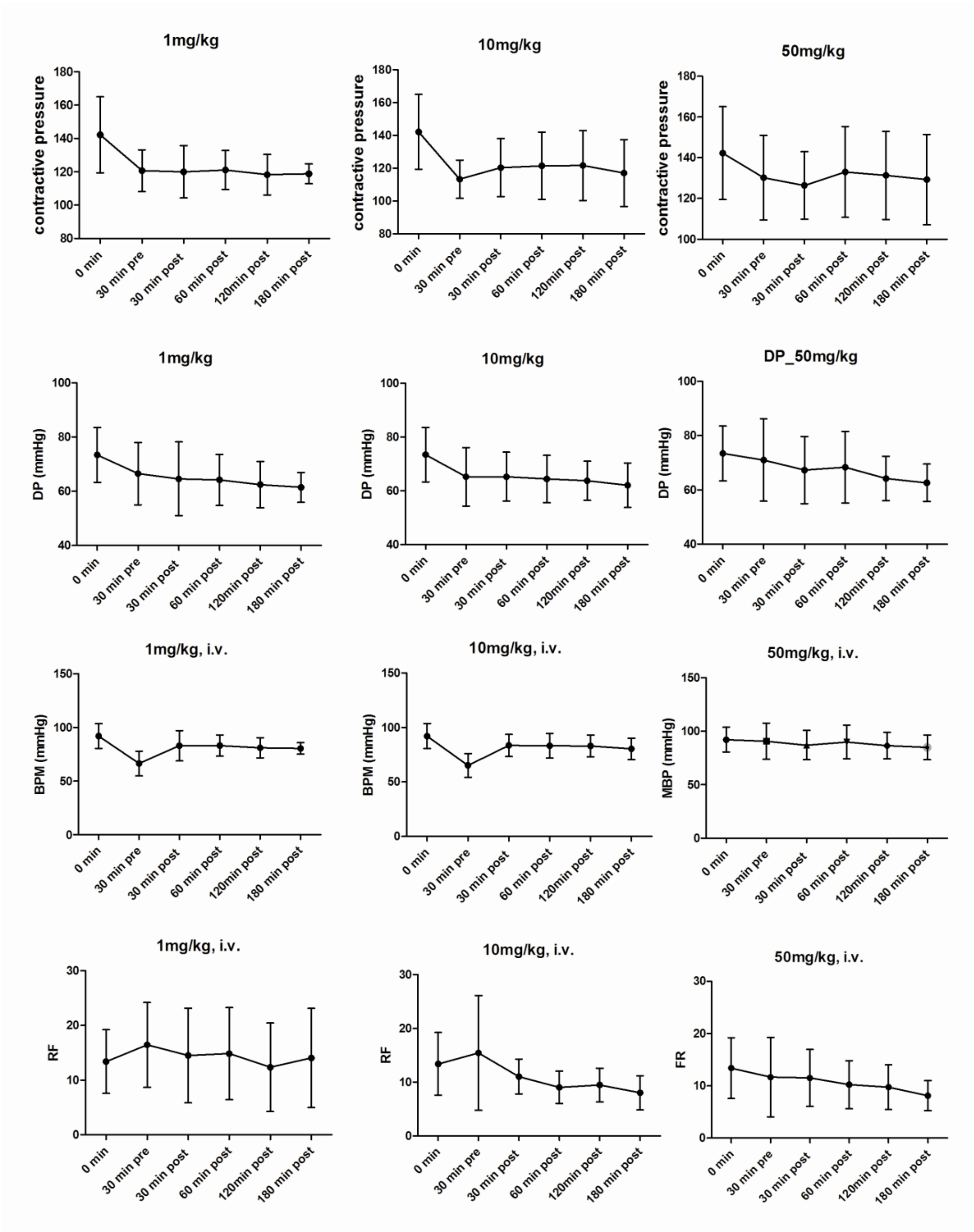

Supplementary Figure S3. Effects of Eu1.6 on cardiac and respiratory function of dogs. For all parameters monitored (**a-d**), no significant changes were observed in animals that received Eu1.6 at three doses tested compared to the animals in vehicle-controlled group ($p > 0.05$, t-test). All animals ($n = 24$) also showed no obvious change after drug administration as evidenced by pre- and post-dose being similar. This suggests that Eu1.6 has no significant effect on the cardiac and respiratory function at the doses tested. Data represent the mean ± SD.

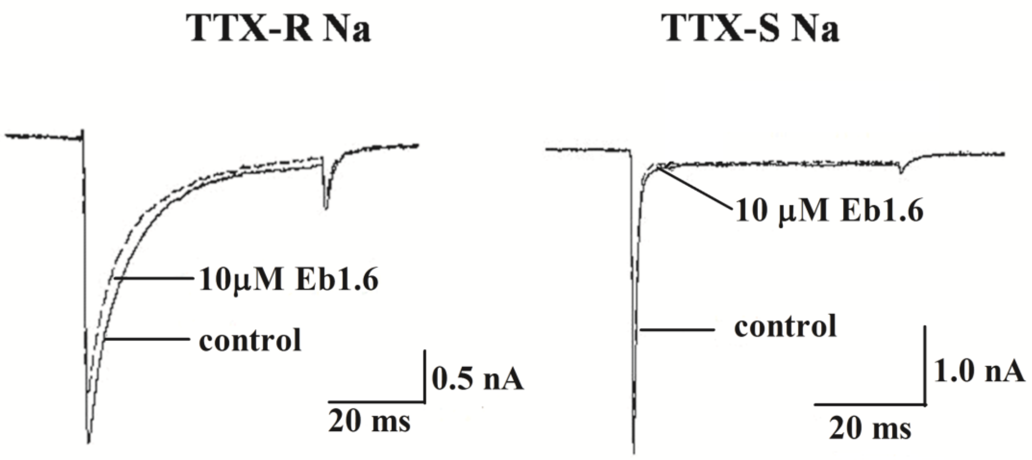

Supplementary Figure S4. Effect of Eu1.6 on voltage-gated sodium currents in rat DRG neurons. TTX-S and TTX-R Na^+ currents were evoked by step depolarization (50 ms) to −10 mV and +10 mV, respectively, from a holding potential of −80 mV. Eu1.6 (10 µM) inhibited TTX-S and TTX-R Na^+ currents by 5.5 ± 2.1% and 16.5 ± 4.2% (n = 4-6 for each), respectively, suggesting that Eu1.6 weakly inhibited TTX-R Na^+ channels but was relatively inactive on TTX-S Na^+ channels in rat DRG neurons ($p < 0.05$, t-test)

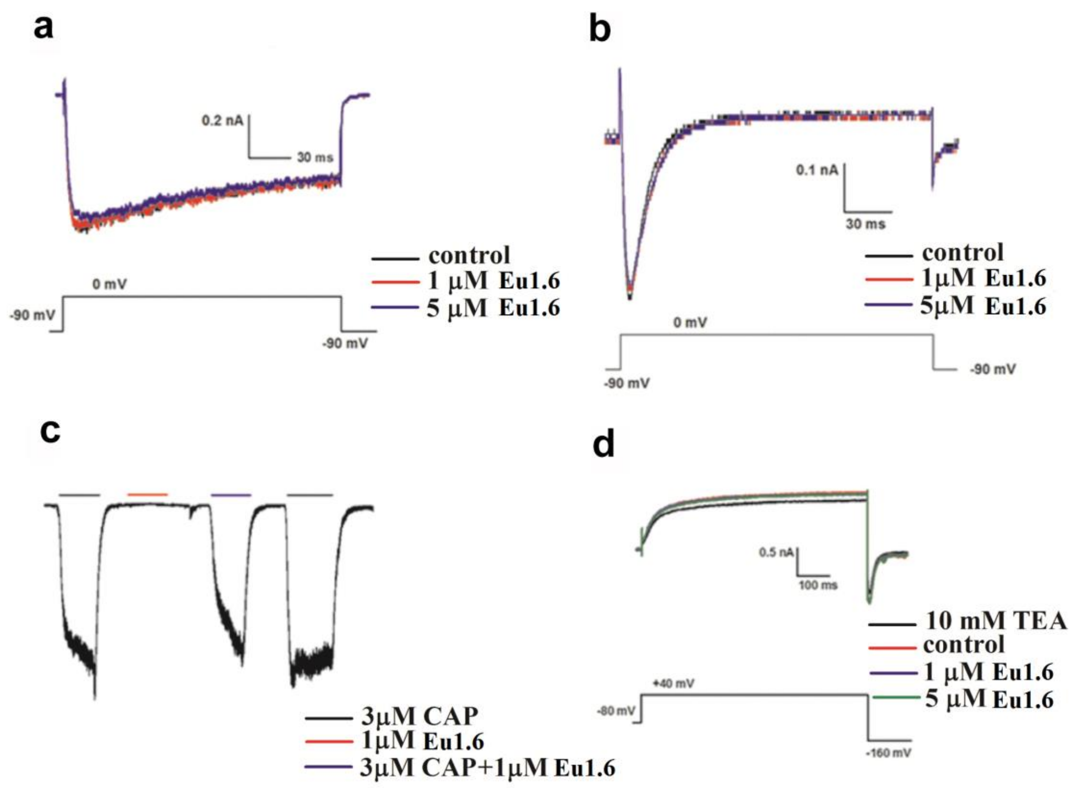

Supplementary Figure S5. Effect of Eu1.6 on L- and T-type calcium channels, VR1 and KCNQ1 expressed in HEK293 cells. Recording from HEK293 cells transfected with **(a)** L-type, **(b)** T-type calcium channels, **(c)** VR1 channels and **(d)** KCNQ1 channels (n = 4-6 for each) shows that 1-5 µM Eu1.6 did not affect these channels current. Any inhibition observed for the above channels in the presence of 5 µM Eu1.6 was < 5% ($p > 0.05$, t-test). Inserts below current traces show the voltage pulse protocol for channel activation (**a,b,d**) whereas VR1 channels (**c**) were activated by capsaicin (CAP).

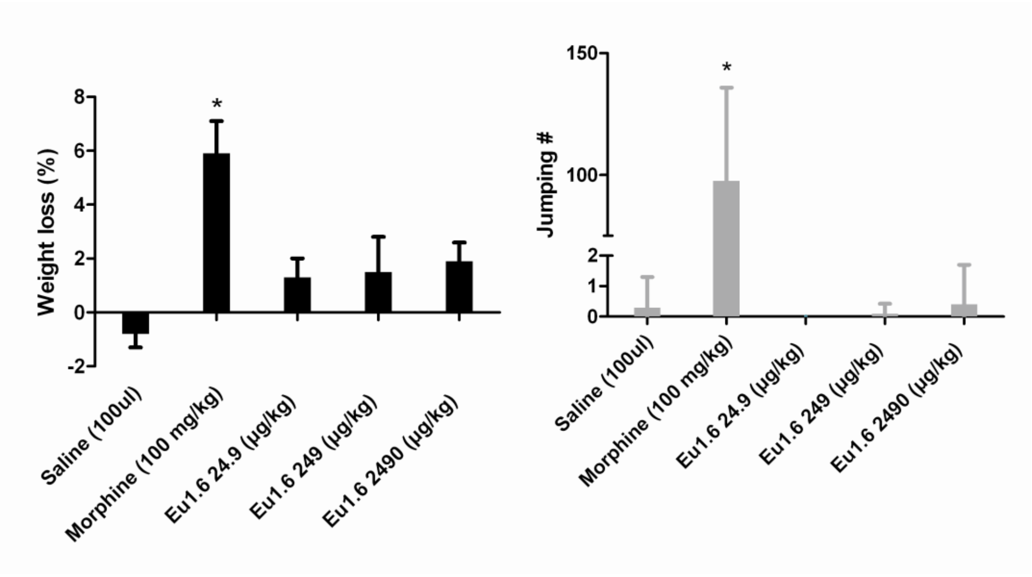

Supplementary Figure S6. Eu1.6 did not produce morphine-like dependence. Bar graphs depict the naloxone-induced jumping number (left) and weight loss (right) before and after the administration of naloxone. Eu1.6 did not cause morphine-like dependence and apparent weight loss, even though the dose of Eu1.6 used in this experiment is 100-fold higher than that used in the analgesic experiments using the same administration route (n = 10 per group).

Supplementary References

1 Malmberg, A. B., Gilbert, H., McCabe, R. T. & Basbaum, A. I. Powerful antinociceptive effects of the cone snail venom-derived subtype-selective NMDA receptor antagonists conantokins G and T. *Pain* **101,** 109-116 (2003).

2 Xiao, C. *et al.* NR2B-selective conantokin peptide inhibitors of the NMDA receptor display enhanced antinociceptive properties compared to non-selective conantokins. *Neuropeptides* **42,** 601-609, doi:10.1016/j.npep.2008.09.003 (2008).

3 Van Deuren, B. *et al.* The fentanyl/etomidate-anaesthetised beagle (FEAB) dog: a versatile in vivo model in cardiovascular safety research. *J Pharmacol Toxicol Methods* **60,** 11-23, doi:10.1016/j.vascn.2009.04.195 (2009).

4 Li, W. *et al.* Highly selective and potent μ opioid ligands by unexpected substituent on morphine skeleton. *Bioorg Med Chem Lett* **20,** 418-421, doi:10.1016/j.bmcl.2009.07.119 (2010).

5 Liu, J. G. & Prather, P. L. Chronic exposure to μ-opioid agonists produces constitutive activation of μ-opioid receptors in direct proportion to the efficacy of the agonist used for pretreatment. *Mol Pharmacol* **60,** 53-62 (2001).

6 Xiao, Y. C. & Liang, S. P. Inhibition of sodium channels in rat dorsal root ganglion neurons by Hainantoxin-IV, a novel spider toxin. *Sheng Wu Hua Xue Yu Sheng Wu Wu Li Xue Bao (Shanghai)* **35,** 82-85 (2003).

7 Wang, H. *et al.* The venom of the fishing spider *Dolomedes sulfurous* contains various neurotoxins acting on voltage-activated ion channels in rat dorsal root ganglion neurons. *Toxicon* **65,** 68-75, doi:10.1016/j.toxicon.2013.01.014 (2013).

8 Liu, R. *et al.* Human β-defensin 2 is a novel opener of Ca^{2+}-activated potassium channels and induces vasodilation and hypotension in monkeys. *Hypertension* **62,** 415-425, doi:10.1161/HYPERTENSIONAHA.111.01076 (2013).

9 Gan, G. *et al.* Structural basis for toxin resistance of β4-associated calcium-activated potassium (BK) channels. *J Biol Chem* **283,** 24177-24184, doi:10.1074/jbc.M800179200 (2008).
